# Silver and ultrasmall iron oxides nanoparticles in hydrocolloids: effect of magnetic field and temperature on self-organization

**DOI:** 10.1038/s41598-018-22426-2

**Published:** 2018-03-06

**Authors:** Olena Ivashchenko, Barbara Peplińska, Jacek Gapiński, Dorota Flak, Marcin Jarek, Karol Załęski, Grzegorz Nowaczyk, Zuzanna Pietralik, Stefan Jurga

**Affiliations:** 1 NanoBioMedical Centre, Adam Mickiewicz University in Poznań, 61614 Poznań, Poland; 2 Department of Molecular Biophysics, Adam Mickiewicz University in Poznań, 61614 Poznań, Poland; 3 Department of Macromolecular Physics, Adam Mickiewicz University in Poznań, 61614 Poznan, Poland

## Abstract

Micro/nanostructures, which are assembled from various nanosized building blocks are of great scientific interests due to their combined features in the micro- and nanometer scale. This study for the first time demonstrates that ultrasmall superparamagnetic iron oxide nanoparticles can change the microstructure of their hydrocolloids under the action of external magnetic field. We aimed also at the establishment of the physiological temperature (39 °C) influence on the self-organization of silver and ultrasmall iron oxides nanoparticles (NPs) in hydrocolloids. Consequences of such induced changes were further investigated in terms of their potential effect on the biological activity *in vitro*. Physicochemical characterization included X-ray diffraction (XRD), optical microscopies (SEM, cryo-SEM, TEM, fluorescence), dynamic light scattering (DLS) techniques, energy dispersive (EDS), Fourier transform infrared (FTIR) and ultraviolet–visible (UV-Vis) spectroscopies, zeta-potential and magnetic measurements. The results showed that magnetic field affected the hydrocolloids microstructure uniformity, fluorescence properties and photodynamic activity. Likewise, increased temperature caused changes in NPs hydrodynamic size distribution and in hydrocolloids microstructure. Magnetic field significantly improved photodynamic activity that was attributed to enhanced generation of reactive oxygen species due to reorganization of the microstructure.

## Introduction

Nowadays, the researchers’ interest is moving from “single” nanoparticles (NPs) to micro/nanostructures that can be formed due to the ability of NPs to self-organize. This direction in the evolution of nanotechnology appeared simultaneously in different fields of nanoscience, exemplified by self-organizing polymers and metal oxides, three dimensional (3D) graphene architectures and metamaterials^1–4^. 3D micro/nanostructures assembled from various nanosized building blocks are of great scientific interests due to their combined features in the micro- and nanoscale. They are extensively applied in catalysis, environment and new energy sources^5,6^.

The ability of iron oxide NPs to self-organize into flowerlike structures was reported previously, showing their improved ability to remove pollutants from water^2^. Another example of this phenomenon is ferrofluid (dispersion of magnetic iron oxide NPs (≥10 nm)) that becomes strongly magnetized in the presence of a magnetic field. The microstructure of ferrofluids is known to have a complex topology (needles, chains, columns, sheets, lamellar, etc.), however, it’s still under the study, concerning its effect on the physico-chemical properties. Ferrofluids have a wide range of potential application in biomedicine and technology^7–9^. As for the ultrasmall iron oxide NPs dispersion, in the available literature there are no reports devoted to their microstructure and behaviour under applied magnetic field.

Iron oxides in the form of magnetite or maghemite NPs due to their biocompatibility, chemical stability and magnetic properties are widely studied for their use in biomedicine^10,11^. However, the influence of their self-organizing microstructure on biomedical interactions has not been studied so far. The properties of such micro/nanosystems might be quite different from those composed of unstructured NPs and may lead to unexpected results. To our knowledge, this aspect is rarely taken into account.

A combination of magnetic iron oxide with silver NPs allows to obtain nanomaterial with combined properties of its both components: magnetic, optical and antimicrobial. Silver NPs, besides their antimicrobial, antifungal and antiviral activity, enhance optical signals^12,13^. Therefore such a combination is expected to exhibit high potential for biomedicine, in particular as a multimodal material with therapeutic and optical imaging abilities.

Recently, a green approach has been adopted to many synthesis procedures of NPs, particularly for biomedical applications^14,15^. In order to improve NPs biocompatibility, plant extracts are often used instead of toxic chemicals^16,17^. Among the plants, ginger rhizome is of great scientific interest^18^. Besides specific phenolic compound (gingerol), it contains polysaccharides that may form hydrocolloids^19^; the advantage of this property was utilized in this study.

In this study we investigated the influence of external magnetic field on the self-organization process of the mixture of silver and ultrasmall iron oxide (MAg) NPs, namely, on their microstructure, fluorescent and electrical properties.

It is proposed that increase of temperature from 21‒22 °C (room) to 37‒39 °C (human body) can rise the kinetic energy of NPs inducing their Brownian motion, prompting such systems to spontaneous evolution towards thermodynamic equilibrium. This might significantly change the properties of NPs dispersions. In the present study we report on the influence of an increased temperature on NPs hydrodynamic size distribution, microstructure and conductivity.

Further on we investigated the potential of MAg NPs hydrocolloids for photodynamic therapy *in vitro*, taking into account the influence of the aforementioned external factors. The results presented in this study allow for better understanding of the self-organizing behavior of these NPs under applied external conditions, what opens up their new application possibilities.

## Results

### Physicochemical characterization of USIO, Ag and MAg NPs

#### SEM EDS measurements

The results of SEM EDS measurements revealed (see Table 1 and Supplementary Fig. S1) that USIO NPs are mainly composed of Fe and O. Besides that there were admixtures of C, Na and Mg originating from the synthesis. Ag NPs contain approximately 90 wt% of Ag and admixtures (C, O, Mg). MAg NPs are mainly composed of Fe, O and Ag. The content of Ag in MAg samples differs approximately by a factor of two (8.5 and 14.9 wt%) so this feature is used for appellation of the MAg samples: Ag 8% and Ag 15%, respectively. MAg NPs also contain admixtures (C, Na, Mg).

**Table 1:** Elemental composition of USIO, Ag and MAg NPs according to SEM EDS measurements.

	Elemental content, wt%			
	USIO NPs	Ag NPs	MAg (Ag 8%)	MAg (Ag 15%)
C	6.0 ± 0.8	4.5 ± 0.5	4.6 ± 1.1	5.0 ± 1.3
O	46.4 ± 1.8	4.7 ± 0.6	38.7 ± 6.9	38.8 ± 4.8
Na	5.9 ± 0.8	—	4.7 ± 1.1	3.7 ± 0.5
Mg	0.6 ± 0.1	1.2 ± 0.2	0.7 ± 0.1	0.7 ± 0.1
Fe	41.0 ± 2.1	—	42.7 ± 8.3	37.0 ± 5.2
Ag	—	89.6 ± 1.0	8.5 ± 0.9	14.9 ± 1.7

#### High resolution TEM and SEM measurements

**Figure 1:**
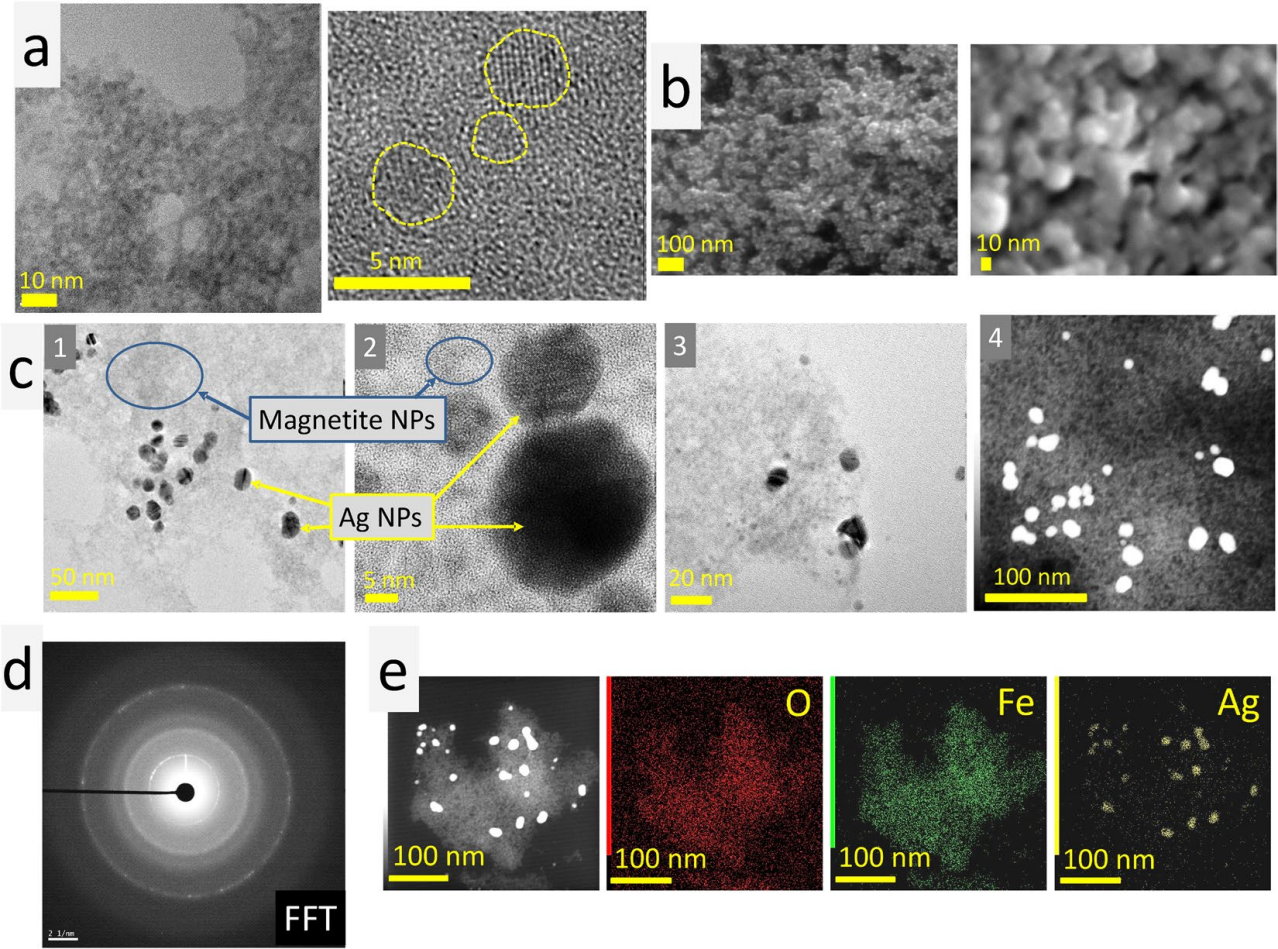
TEM images of USIO NPs (**a**); SEM images of Ag NPs (**b**); TEM images of MAg NPs (**c**): 1 and 2 (bright field) correspond to Ag 8%, 3 (bright field) and 4 (dark field) - to Ag 15%; FFT analysis of iron oxide NP in MAg (**d**); TEM EDS elemental mapping of MAg (Ag 8%) (**e**).

Investigation of NPs morphology by means of TEM and SEM microscopies showed that USIO NPs are ~3 nm in size; atomic resolution image revealed their crystalline form (Fig. 1a). Ag NPs are roundish, 10‒30 nm in size (Fig. 1b). MAg NPs are composed of two types of NPs: USIO (~3 nm) and Ag (10‒20 nm) (Fig. 1c). A fast Fourier transformation (FFT) analysis proved that iron oxide NPs exhibit highly crystalline characteristic (Fig. 1d). D-spacing of planes (311), (400) and (440) is similar to those of magnetite or maghemite^20^, however, due to the relatively low resolution of FFT image it’s difficult to identify iron oxides phases unambiguously. Figure 1e presents typical result of TEM EDS elemental analysis for MAg NPs showing distribution of Fe, Ag and O elements.

#### XRD results

**Figure 2:**
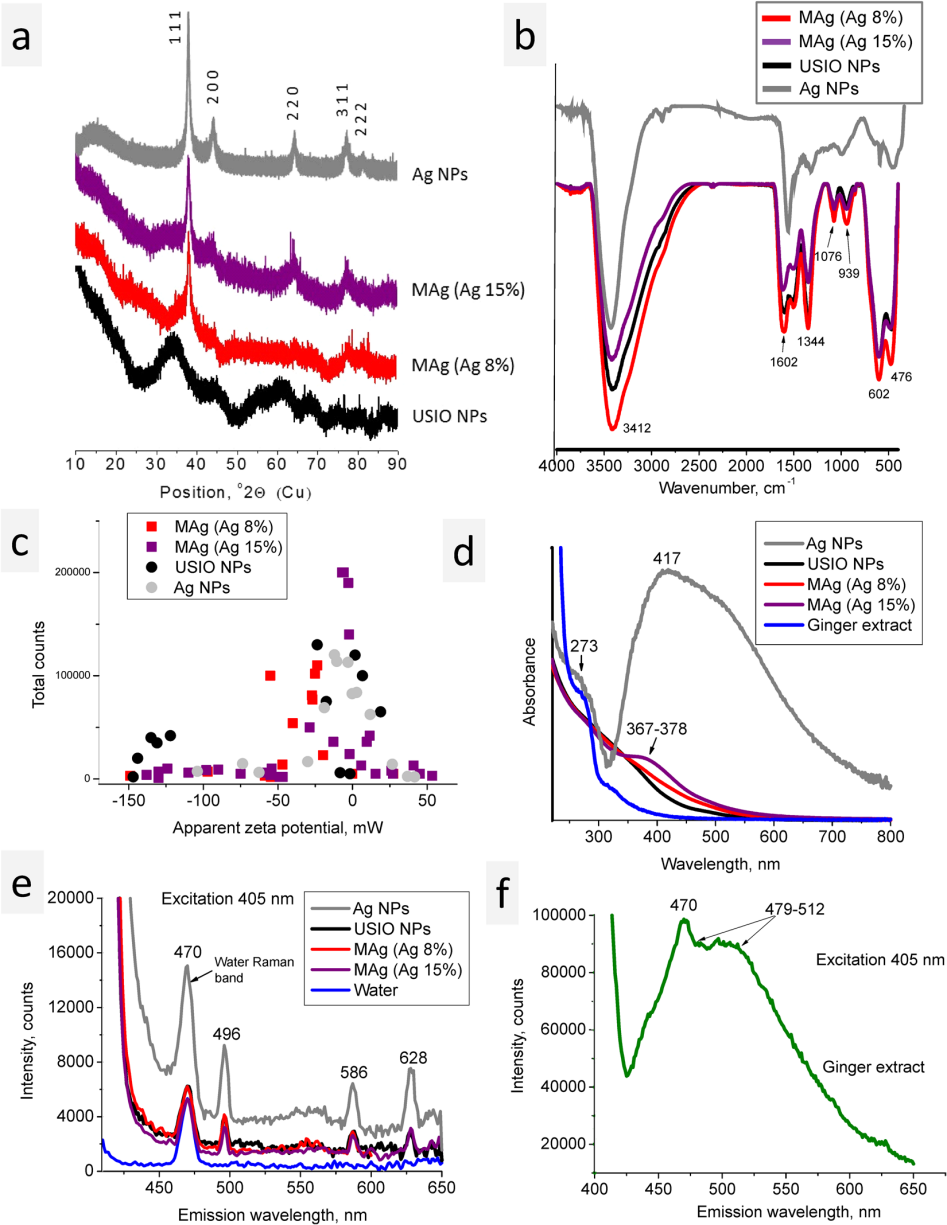
XRD diffractograms (**a**), FTIR spectra (**b**); zeta potential variety of USIO, Ag and MAg NPs (**c**); UV-Vis spectra of ginger, USIO, Ag and MAg NPs (**d**); fluorescence emission spectra of USIO, Ag and MAg NPs dispersions (**e**) and ginger extract (**f**).

XRD diffractogram of USIO NPs showed several broad peaks, at 34‒36, 44‒45, 60‒63 and 67‒70 °2Theta, which may be attributed to both magnetite or maghemite phases^20^ (Fig. 2a). The broadness of the peaks made the precise phase composition analysis difficult. The same problem was reported for magnetite NPs with similar size (~4 nm)^21^. Simulation of Sherrer broadening for NPs size from 2.5 to 13.4 nm clearly showed that ultrasmall size of NPs drastically influences the broadness of diffraction peaks^22^. In addition, the surface of magnetite NPs differs from the volume due to the oxidation processes resulted in transition to maghemite phase^23^; so the term “iron oxide” is used through the article.

XRD diffractogram of Ag NPs revealed the presence of broad peak at 14.50 °2Theta and sharp peaks (38.1, 44.3, 64.5, 77.6 and 81.6 °2Theta). The peak at 14.5 °2Theta points to a presence of organic compounds originating from ginger, the other peaks are related to silver^20^.

XRD diffractograms of MAg NPs revealed four peaks (38.2, 44.5, 64.4 and 77.6 °2Theta) attributed to silver; lack of iron oxide diffraction peaks may be partially related to overlap of their peaks by silver ones.

#### FTIR measurements

FTIR spectra of USIO and MAg NPs are similar (Fig. 2b); they reveal peaks at 476 and 602 cm^−1^ (Fe-O vibrations, stretching and torsional modes)^24^. The peaks at 939, 1076, 1344 and 1602 cm^−1^ (C‒O, C‒N stretching, C‒H bending vibrations in amino groups) point to a presence of organic compounds on the surface of NPs originated from ginger. The comparison with ginger spectrum^22^ showed that these peaks are shifted, which suggests chemical bonding of these compounds. A broad peak centred at 3412 cm^−1^ is ascribed to O‒H groups^24,25^.

FTIR spectrum of Ag NPs revealed the peaks at 400‒800, 1091, 1400 and 1637 cm^−1^ which pointed to a mineral and organic compounds originated from ginger. Comparison with ginger spectrum^22^ revealed difference in the peaks intensity ratio and a shift of their positions that pointed to a selective adsorption and chemical bonding to the surface of Ag NPs.

#### Zeta potential measurements

Measurements of zeta potential give information about stability of NPs dispersions (the higher zeta potential the higher stability)^26^ and possibility for electrostatic interaction (Table 2).

**Table 2:** Mean zeta potential of NPs dispersions.

Sample	Mean zeta potential, mV
Ag NPs	−8.4 ± 0.4
USIO NPs	−31.2 ± 1.9
MAg (Ag 8%)	−26.4 ± 1.9
MAg (Ag 15%)	−12.6 ± 3.8

Zeta potential of Ag NPs pointed to instability of their dispersions; for USIO NPs it correlates with high stability of their dispersions. Zeta potential of MAg NPs dispersions had an intermediate value, in spite of the fact that their dispersions were very stable. This discrepancy was explained by variety of zeta potential values (Fig. 2c): some flocculates possessed charge that differs significantly from mean values (e.g., −150 and 50 mV). The serial measurements of MAg dispersions revealed dynamic changes in charge distribution (see Supplementary Fig. S2), whereas the mean value remained the same. These results pointed to existence of dynamic aggregation-disaggregation processes based on electrostatic interaction between NPs.

#### UV-Vis measurements

UV-Vis spectrum of ginger contains two absorption bands (268‒274, 314‒320 nm) (Fig. 2d). The USIO NPs absorb light within 200‒550 nm. The spectrum of Ag NPs is characterised by strong absorption within 356–559 nm (maximum at ~417 nm) and 268‒274 nm. The latter coincides with the ginger peak and may point to a presence of ginger compounds on the Ag NPs surface, whereas the former is typical for Ag NPs due to the surface plasmon resonance effect^27^. The maximum band position and width of this band depend on NPs size and aggregation process; the maximum at 417 nm is inherent to 8‒10 nm Ag NPs, whereas asymmetrical shape of the band is typical for aggregation^28–31^. The UV-Vis spectra of MAg NPs are similar to USIO NPs spectrum but with low-intensive band at 368‒378 nm related to Ag NPs. This band is shifted to lower value in comparison with that of Ag NPs. In similar systems, such a shift is explained by charge transfer effect occurred due to the difference in electron work function of two metals^27,32–35^. For MAg NPs, the shift may also be explained by charge transfer effect occurred between Ag and iron oxide NPs.

#### Fluorescence measurements

Fluorescence measurements of USIO, Ag and MAg water dispersions: The excitation at 405 nm was chosen due to its frequent application in photodynamic therapy. The spectra of USIO, Ag and MAg NPs revealed three emission bands (496, 586 and 628 nm) (Fig. 2e). The peak at 470 nm is due to water Raman scattering^36^. From the literature data, the peaks at 491 and 486 nm were detected for Ag NPs (excitation 416 and 408 nm, respectively)^37,38^. In our case, USIO NPs also contain these emission bands. As known, when a sample is illuminated by a laser, both photoluminescence and Raman scattering can occur. In order to dissociate photoluminescence from Raman scattering, emission spectra at excitation 400 nm were recorded. At such a negligible shift in excitation wavelengths (Δ5 nm) fluorescence peaks would not change their positions, whereas Raman bands would be shifted. The results showed that all the peaks were shifted pointed to Raman scattering (see Supplementary Fig. S3). Emission spectrum of ginger revealed broad peak within 475‒650 nm (Fig. 2f). Hence, we supposed that the bands of USIO, Ag and MAg NPs are due to Raman scattering of adsorbed ginger compounds.

Fluorescence measurements of USIO, Ag and MAg hydrocolloids: In our previous study we found that MAg NPs in the form of powder or hydrocolloid emit fluorescence when excited at 405 or 780 (two-photon) nm wavelengths, with maximum band position within 458–537 and 650–680 nm, respectively^22^. We established that the first peak is due to ginger compounds, whereas the latter was supposed to be due to surface plasmon resonance of Ag NPs. In this study we aimed to confirm this supposition and identify the contribution of each MAg component to fluorescence spectrum.

Fluorescence images and spectra of the hydrocolloids are shown in Supplementary Fig. S4. As seen, fluorescence emittance of USIO NPs occurred only at 405 nm excitation wavelengths, and may be related to fluorescence of ginger compounds adsorbed on NPs surface^22^. USIO NPs exhibit the lowest fluorescence intensity of the samples. Ag NPs exhibit the fluorescence emittance at two excitation wavelengths (405 and 780 (two-photon) nm). Its wide peak with maximum at ~ 450 nm is due to surface-enhanced fluorescence of ginger compounds, whereas the peak with maximum at ~670 nm is due to surface plasmon resonance effect^39^. The intensity of Ag NPs fluorescence is the highest among the studied samples. MAg hydrocolloids emit fluorescence at both excitation wavelengths and its intensity increases with increase of silver content, which is predictable. These and our previous results cleared up the origin of fluorescence emittance in MAg NPs: surface-enhanced fluorescence of ginger compounds and surface plasmon resonance of Ag NPs.

**Figure 3:**
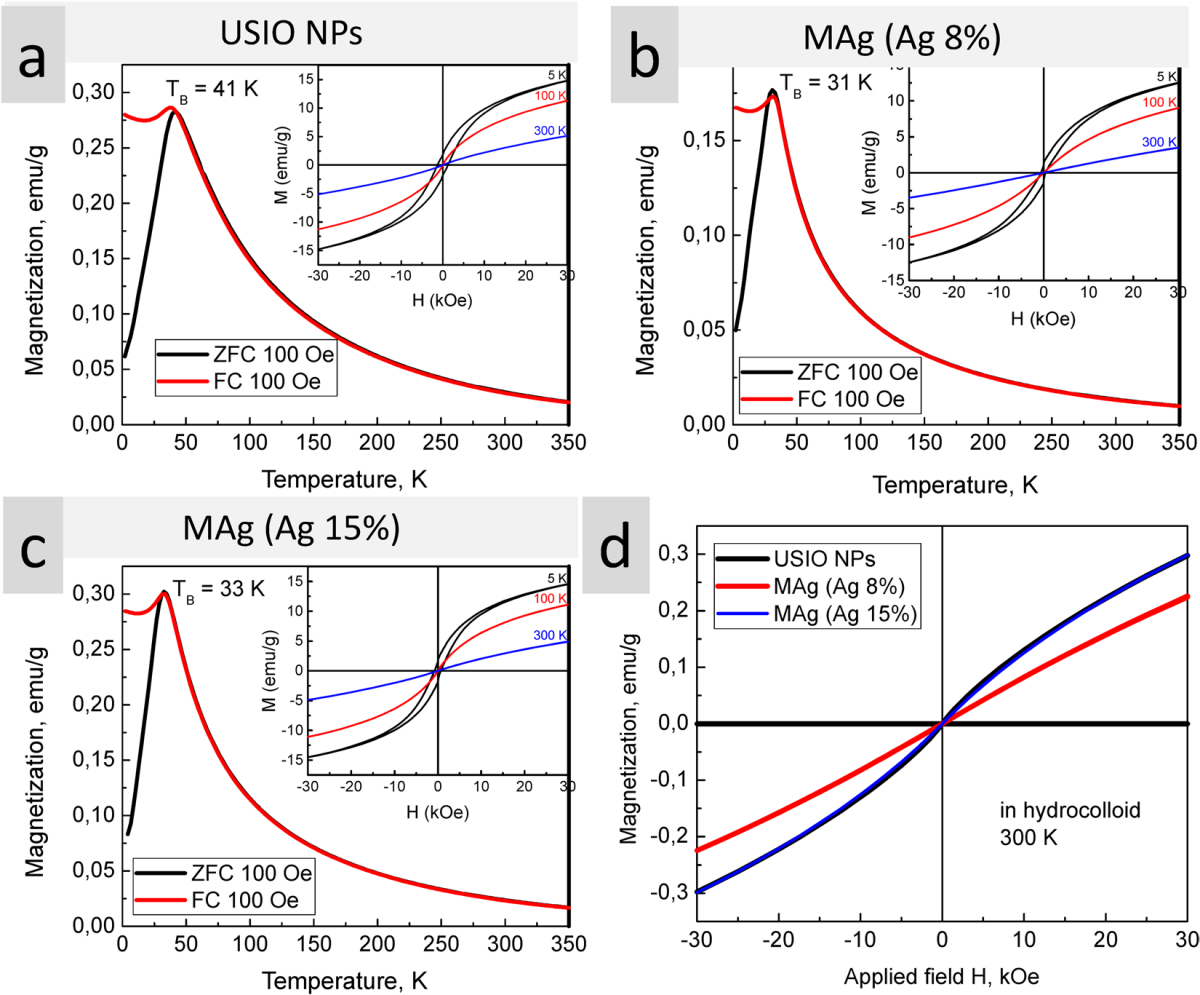
Magnetization measurements: ZFC and FC curves and magnetic field dependence (inset) for USIO and MAg NPs powders (**a**–**c**); magnetic field dependence for USIO and MAg NPs hydrocolloids (**d**).

Magnetization measurements: The zero-field-cooled (ZFC) and field-cooled (FC) magnetization of MAg powders and hydrocolloids were measured at 100 Oe (Fig. 3a–c). For powders, the ZFC and FC curves bifurcate at a certain temperature (T_*B*_) showing a peak at 41 K (USIO NPs) and 31–33 K (MAg NPs). Similar blocking temperature (37 K) was observed for USIO NPs with superparamagnetic properties^40^. As known, the dynamic behaviour of NPs under magnetization is extremely sensitive to particle size distribution: rapid increase of the signal up to T_B_ is characteristic for narrow size distribution; T_B_ shifts to high values with particle size^41,42^. Hence, USIO and MAg NPs have a narrow size distribution.

The magnetic field dependence of magnetization for the powders is presented in Fig. 3a–c (inset). S-shaped dependence with no saturation and no remanence at 100 and 300 K, but with hysteresis loop at 5 K, is characteristic for superparamagnetic behaviour^43,44^. As known, when the size of NPs is close to superparamagnetic critical diameters (for Fe_3_O_4_ ~4 nm), then the magnetic moment is not stable, and therefore H_c_ = 0^43^. This effect is related to (i) high magnetic anisotropy originating from the disordered surface layer and (ii) small apparent magnetic size of the NPs (*d*_0_ ~ 0.56 nm)^40,43^.

The magnetic field dependence of magnetization for the hydrocolloids at 300 K is presented in Fig. 3d. Absence of coercive field and remanent magnetization conforms to superparamagnetic behaviour which is typical for USPIO fluids or ferrofluids^40,44^. Magnetization values are lower than that for ferrofluids^45,46^, but higher than for USIO NPs dispersed in organic fluid^40^. Calculation of magnetization values in hydrocolloids relative to NPs concentration gave the same results (±relative error) as for powders (see Supplementary Fig. S5).

### Influence of magnetic field on USIO and MAg NPs hydrocolloids

We have previously discovered the highly-ordered microstructure in MAg NPs hydrocolloids^22^. Silver and iron oxide NPs could not be separated from those hydrocolloids by using magnetic field, and no visible changes were observed under the action of magnetic field (in contrast to the typical behaviour of ferrofluids). As the NPs revealed superparamagnetic properties, we hypothesized that it would be possible to influence their structuration by applying magnetic field. To check this hypothesis, cryo-SEM, fluorescence spectroscopy, conductivity and electro potential measurements were used.

As for the outer structure, it was similar to that described earlier^22^. Net pattern as well as parallel stripes were typical for the surfaces of all the samples (see Supplementary Fig. S6). It was shown that surface net structure is inherently dependent on the water presence and became visible due to the sublimation process. Water molecules that were not H-bonded evaporated faster than those H-bonded. This structure was supposed to be responsible for surface tension.

**Figure 4:**
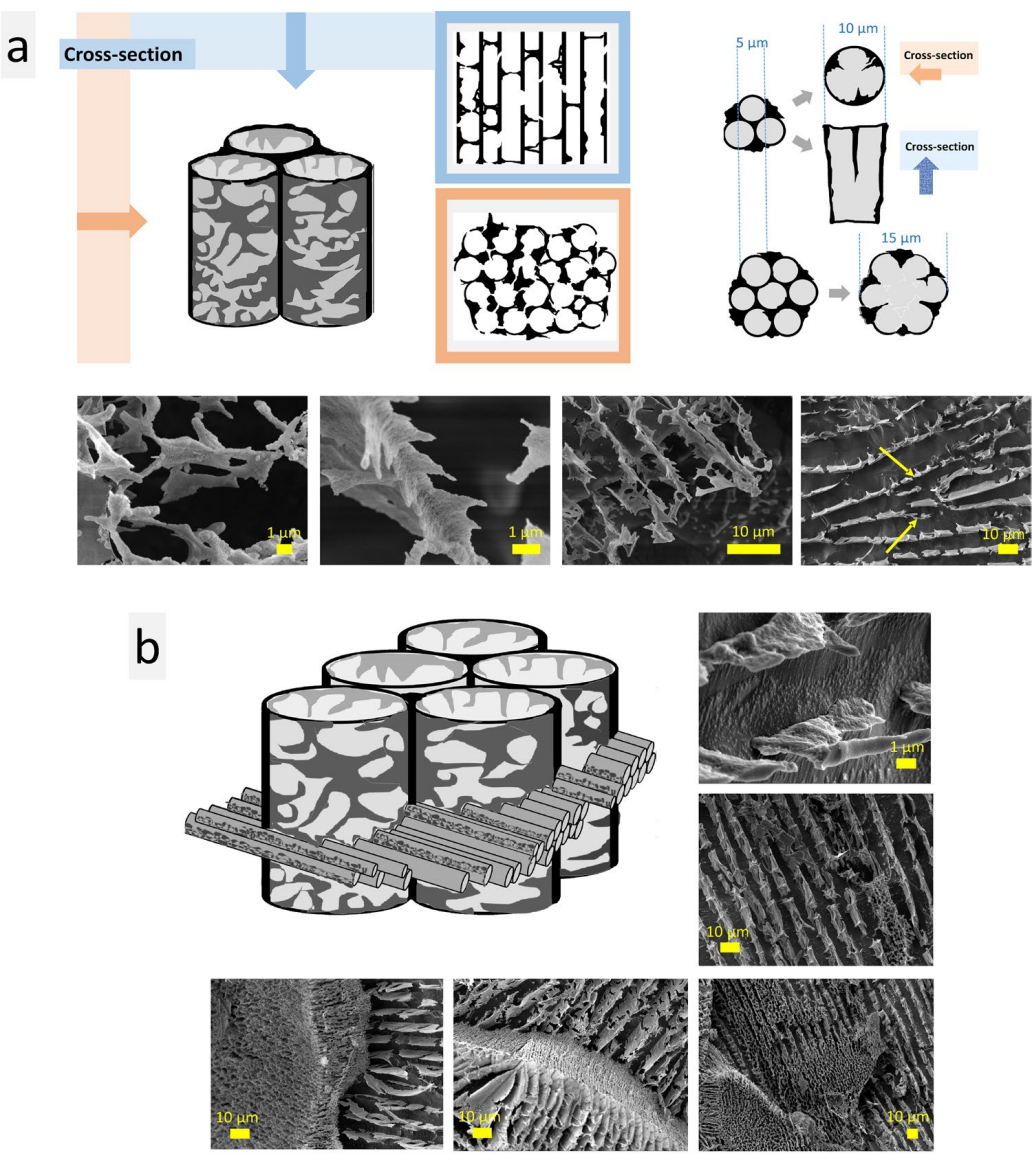
Schemes and representative cryo-SEM images of microstructure elements - porous tubes (**a**) observed in USIO and MAg hydrocolloids (yellow arrows pointed to merged tubes); the porous tubes coexist with smaller ones (<5 µm in diameter) oriented perpendicularly (**b**).

For the inner structure, highly-oriented parallel stripes and sponge structures were typical. Parallel stripes were supposed to be as walls of porous tubes with parallel alignment. Depending on their cross-section direction, we observed parallel stripes or sponge (Fig. 4a, left). Diameter of these tubes was 6–9 µm. Several single tubes could merge forming tubes with larger diameter (Fig. 4a). These porous tubes coexist with smaller ones (<5 µm in diameter) oriented perpendicularly, so that they fill inner volume of larger tubes (Fig. 4b). These complex porous structures form lamellae domains that are oriented in different directions. The size of these domains varies (≤70‒300 µm). The microstructure left a complex patterns when the drop of hydrocolloid was dried (“coffee ring effect”) (see Supplementary Fig. S7).

**Figure 5:**
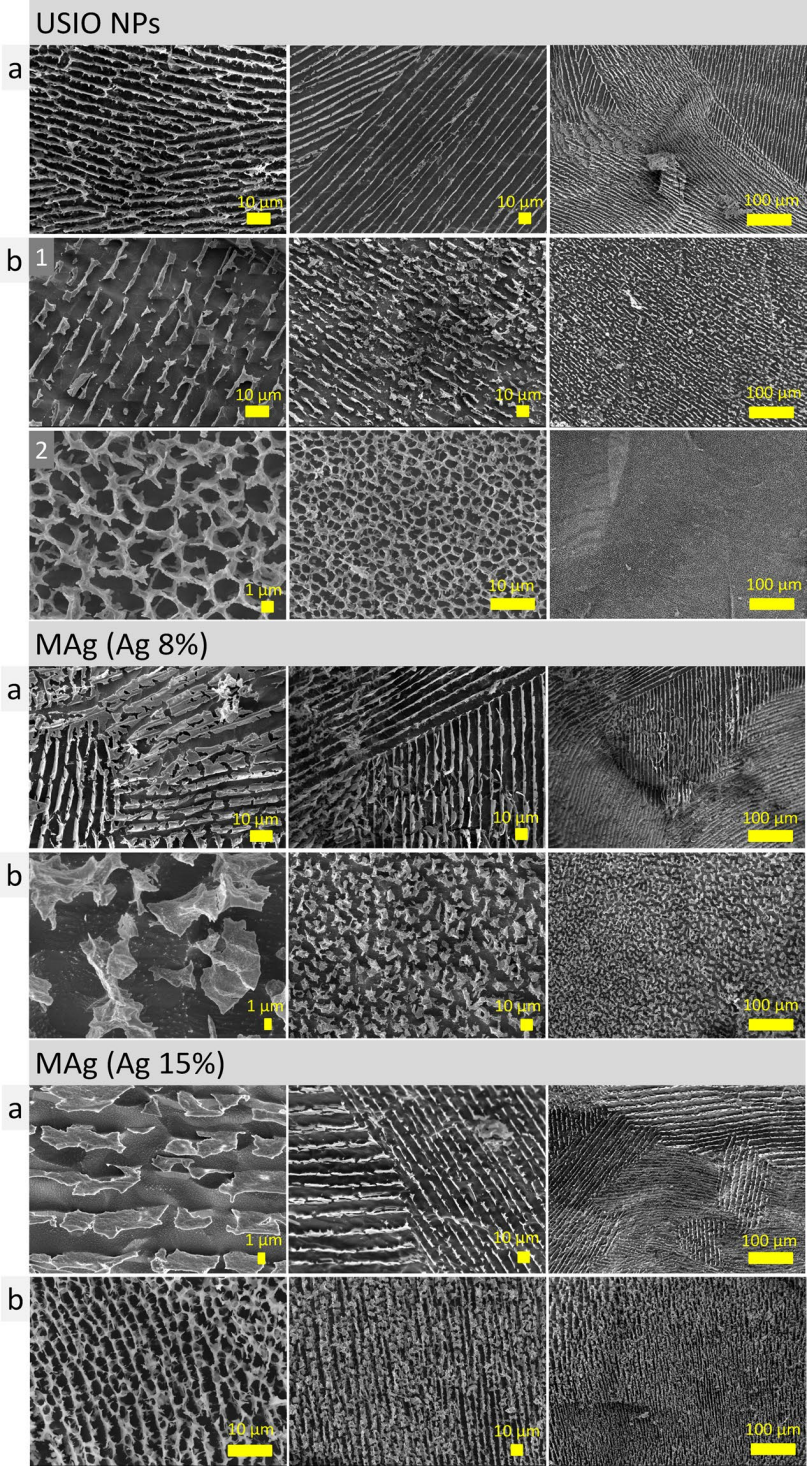
Cryo-SEM images of the USIO and MAg hydrocolloids without (**a**) and with (**b**) exposition under magnetic field. Uniform stripes (**b1**) and sponge (**b2**) structures of USIO hydrocolloids are shown.

For the samples exposed to magnetic field, it was noticed that <5 h exposition caused disintegration of initial microstructure (images not shown), whereas longer exposition (18‒20 h) caused microstructure reorganization (Fig. 5). Magnetic field increased the uniformity of microstructure: instead of differently oriented domains with size range ≤70‒300 µm, a single domain appeared (≫500 µm). Uniformity was observed for both parallel stripes and sponge structures. This observation correlates with previously reported results for ferrofluids, where application of magnetic field caused an increase of microstructure elements size (clusters or chains)^8,9^.

In order to reveal elements distribution in microstructures studied, cryo-SEM EDS mapping before and after exposition to magnetic field were performed (see Supplementary Fig. S8). The microstructure mainly consist of Fe (USIO) and Fe, Ag (MAg), whereas O is predominantly situated between structural elements (the amount of O in water is higher than in iron oxides). Thus, exposition to magnetic field caused reorganization of both type of MAg NPs, superparamagnetic iron oxide and diamagnetic Ag.

**Figure 6:**
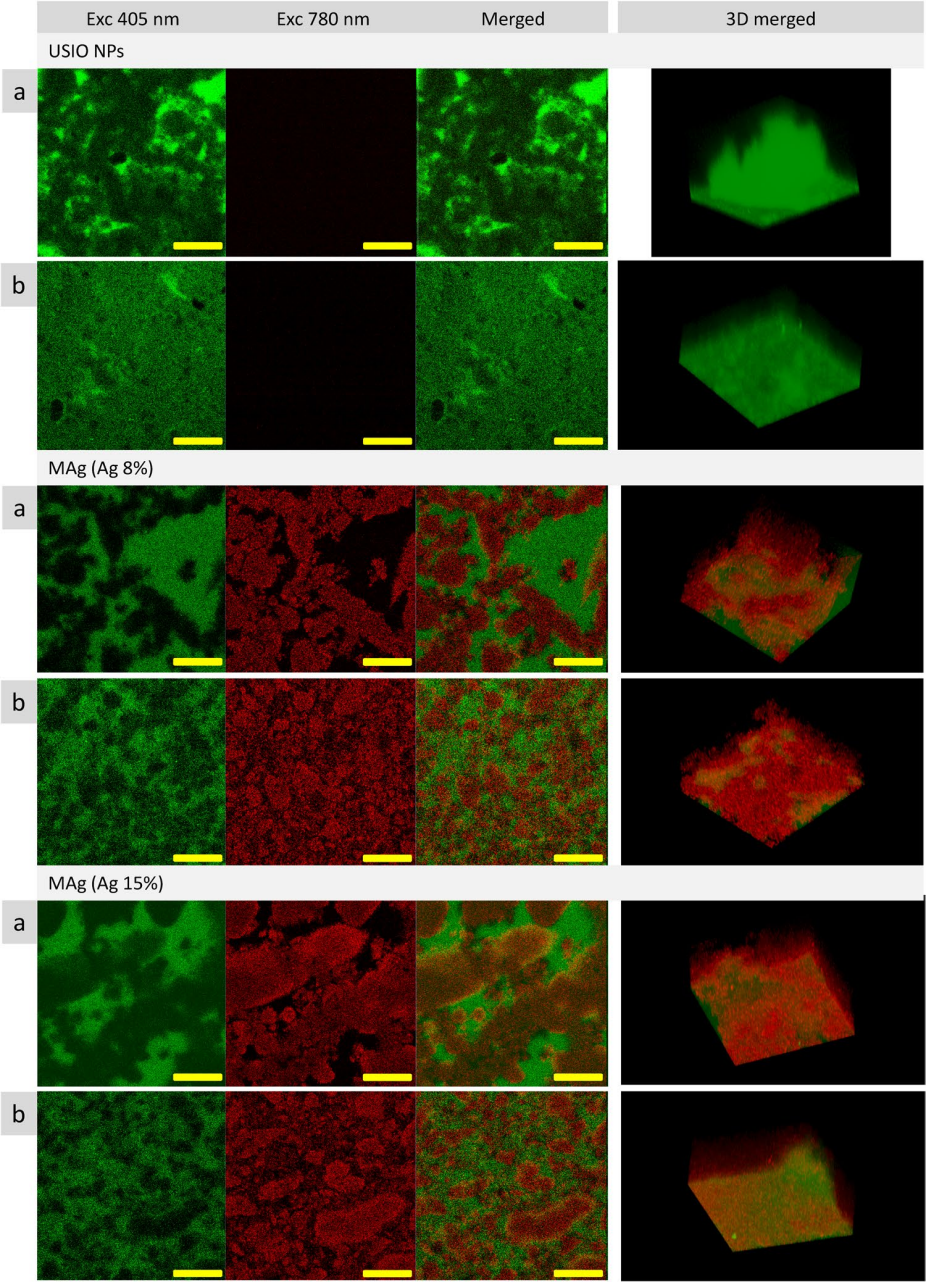
Fluorescence images of the USIO and MAg hydrocolloids without (**a**) and with (**b**) exposition under magnetic field (scale bar 50 µm).

The observed changes in USIO and MAg hydrocolloids microstructure under the action of magnetic field should also be visualized by fluorescence spectroscopy. We previously found^22^ that fluorescent emission is connected with microstructure: different elements emit fluorescence at different excitation and with different intensity. To check this hypothesis, fluorescence images of the hydrocolloids were recorded before and after exposition to magnetic field (Fig. 6). Magnetic field influenced the “topography” of fluorescence emittance in such a way that strongly emitting areas (microstructure elements) became smaller and more homogenously distributed. We also noticed the increase of fluorescence intensity for MAg (Ag 8%) NPs hydrocolloids after exposition to magnetic field (using the same settings as for sample without magnetic field gave overexposed images, so the laser intensity had to be decreased).

It was noticed that exposition to magnetic field caused the separation of water in hydrocolloids (9 ± 4 vol%) that pointed to some compression of hydrocolloids under this condition.

These changes in microstructure of USIO and MAg hydrocolloids might influence their electrical properties. The preliminary measurements showed (see Supplementary Table S1) that conductivity and electro potential increased by 5 ± 0.6% after exposition in magnetic field which further confirmed reorganization of the microstructure.

The results allowed us to conclude that USIO and MAg hydrocolloids are sensitive to the action of magnetic field. Magnetic field induced the microstructure uniformity, fluorescence emittance and conductivity of USIO and MAg hydrocolloids.

### Behaviour of USIO, Ag and MAg NPs hydrocolloids at physiological temperature

For NPs in biomedicine, the working temperature lies in the range 37‒39 °C. However, most studies of NPs are performed at room temperature resulting often in a completely different thermodynamic state of the sample. The interplay of weak forces acting between the complex colloidal particles and water molecules results in a great fragility of the structures on every level of their organization. The softness of the interaction potential makes the system especially susceptible to temperature changes. To check the influence of physiological temperature on the NPs size distribution and hydrocolloids microstructure, a set of measurements was performed.

First, DLS technique was used to measure the NPs size distribution (aggregation/disaggregation processes) in water and BSA solution (see Supplementary Fig. S9). For all NPs water dispersions the temperature increase shifted NPs size distribution towards smaller apparent size. For USIO NPs, the wide double peak (vertex at 40.9 and 234 nm) (NPs agglomerates) decreased and sharp peak at 2.7 nm (single NPs) was shifted to the lower values. For MAg NPs, all the peaks decreased and moved to the lower values. Ag NPs water dispersion was not stable and tends to precipitate at room temperature; appearance of two peaks (15.2 and 260.4 nm) pointed to improved stability at 39 °C. DLS measurements performed at relatively high colloid concentration typically show apparent radii much smaller than the actual ones due to the effect of strong interparticle electrostatic interactions. Nevertheless, our results showed that at 39 °C some disaggregation of USIO and MAg NPs indeed took place, and improved stability of Ag NPs dispersion was achieved.

For NPs dispersions in BSA solution at 39 °C a decrease of NPs apparent size was also observed (see Supplementary Fig. S9b). For USIO and MAg NPs, the apparent size was <7 nm at 21 °C and <3 nm at 39 °C, indicating the absence of agglomerates and high stability of dispersions. As the hydrodynamic radius of BSA protein molecules is 3.5 nm^47^ (see Supplementary Fig. S12), the formation of BSA protective layer on the NPs surface (protein corona) is not very likely. BSA is a well-known stabilizer and protector for proteins against their misfolding. The mechanism of BSA disaggregation properties towards NPs is probably similar to that found for proteins: its chaperone-like activity^48^.

DLS measurements showed that at physiological temperature the dispersibility of NPs generally improved compared to room temperature. Observed shift of their apparent size distribution towards smaller values may be explained in terms of enhancement of repulsive interparticle interactions and chaperone-like activity of BSA molecules.

**Figure 7:**
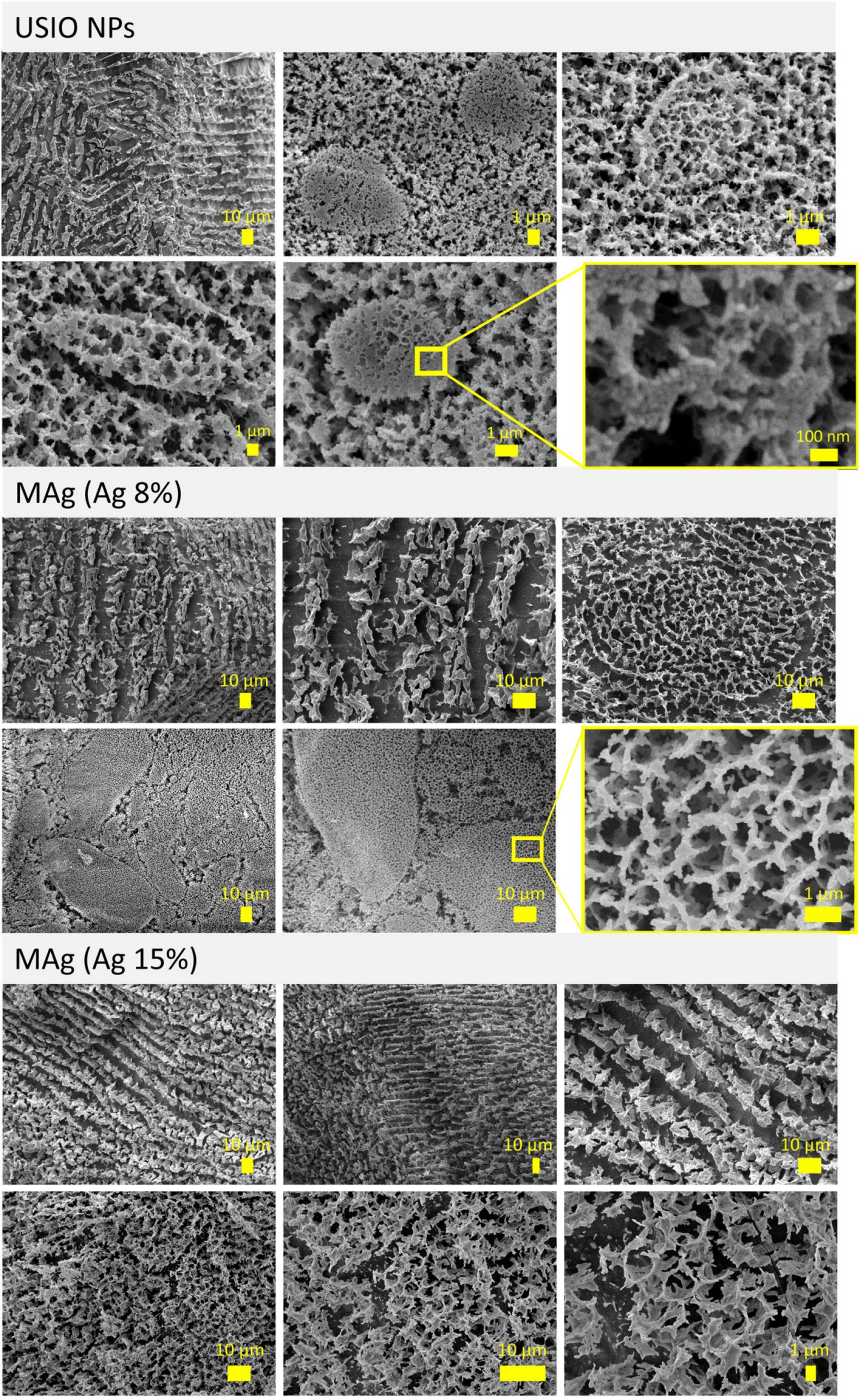
Cryo-SEM images of USIO and MAg hydrocolloids after exposition at 39 °C.

In order to check the influence of physiological temperature on the microstructure of the USIO and MAg hydrocolloids, cryo-SEM measurements were performed (Fig. 7). As seen, exposition to physiological temperature did not destroy the microstructure of hydrocolloids. Lamellae domains and sponge structures were still observed, however, in comparison to the results obtained at room temperature (see Fig. 5a), some differences were found. First, the elements of microstructure became rough. Second, instead of regular geometric shapes, roundish shapes constructed from sponge with pore size in the range 100‒1000 nm appeared. These two features were inherent for all the samples studied.

Such changes in the microstructure could also influence the conductivity and electro potential of the hydrocolloids. Indeed, conductivity of the hydrocolloids increased by ~30%, whereas electro potential values varied within the standard error ±3% (see Supplementary Table S2). Increase of conductivity confirmed changes in hydrocolloids microstructure at physiological temperature.

### Photo-cytotoxicity of USIO and MAg NPs towards HeLa cancer cells – effect of an external magnetic field

**Figure 8:**
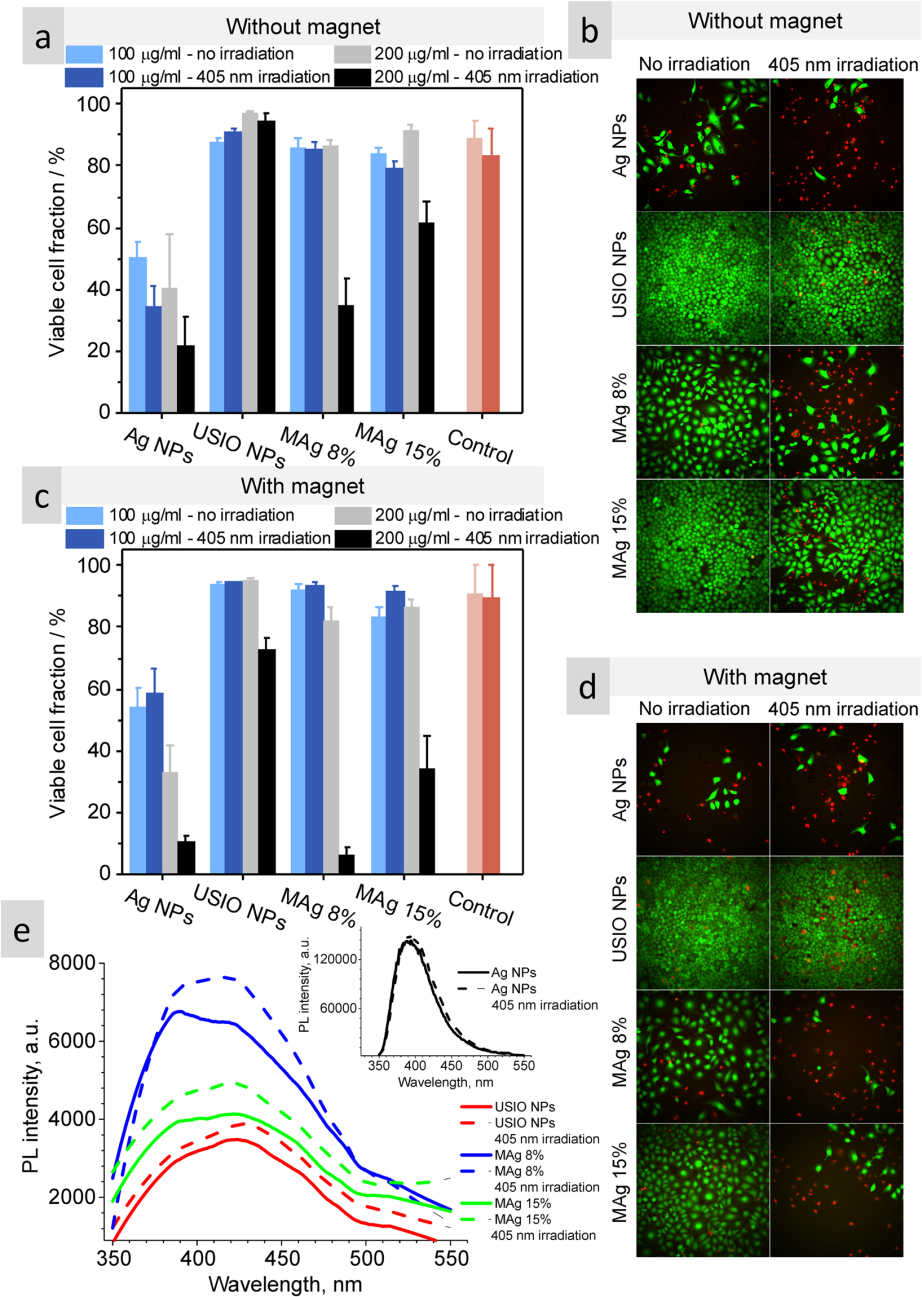
The viability of the irradiated and not irradiated HeLa cells with Ag, USIO and MAg NPs with (**a**,**b**) and without (**c**,**d**) action of magnetic field; fluorescence spectra of non-irradiated and irradiated NPs showing generation of OH^•^ (**e**).

It is known that NPs containing silver has got an increased interest in antitumor photodynamic therapy (PDT), due to photosensitive properties of silver^49,50^. As USIO and MAg NPs hydrocolloids were found to be sensitive to the action of magnetic field and temperature, it was reasonable to assume that these two factors can also affect their biological activity. Therefore, the photo-cytotoxicity of the NPs towards HeLa cancer cells with or without magnetic field exposure was investigated (Fig. 8a–d). Fluorescence images show live (green) and dead (red) cells population (Fig. 8b,d). For the experiment performed without the magnetic field, the viability of the non-irradiated cells exposed to 100 and 200 µg/ml USIO and MAg NPs is at the same level as the viability of control cells, what confirms their biocompatibility. Upon the irradiation the viability of control cells decreased by 5‒9%, whereas the viability of the cells with 100 and 200 µg/ml USIO and 100 µg/ml MAg NPs remained the same (within the standard error), revealing the cytoprotective properties of USIO NPs against irradiation. MAg (Ag 8 and 15%) NPs (200 µg/ml) demonstrated significant phototoxic effect (the viability reduced by 52 and 30%, respectively). Quite expectedly, Ag NPs revealed the highest cytotoxicity among the samples: the viability reduced to 40–50%. Irradiation of the cells with Ag NPs reduced their population to 22–34%.

Concerning the experiment with magnetic field, the viability of the non-irradiated and irradiated cells with USIO and MAg NPs (100 µg/ml) remained at the same level as control. Control cells revealed higher stability to irradiation in comparison to that without magnetic field. USIO NPs (200 µg/ml) revealed weak photo-cytotoxic properties (viability decreased by 21%). MAg (Ag 8% and 15%) NPs exhibited significant photo-cytotoxicity in combination with the action of magnetic field as the viability decreased to 5 and 34%, respectively. MAg (Ag 8%) NPs revealed better photo-cytotoxic effect than MAg (Ag 15%), which is at comparable photo-cytotoxicity level of Ag NPs. It is important that MAg NPs in comparison to Ag NPs do not exhibit dark cytotoxicity.

The results showed that USIO and MAg NPs are biocompatible without irradiation and magnetic field. However, both USIO and MAg NPs were found to be cytotoxic under the action of magnetic field and irradiation. These results demonstrate that magnetic field, changing the microstructure, has also an influence on photo-dynamic reactivity of USIO and MAg NPs at higher concentration.

Ag NPs have been proved to have an anti-tumor activity with an apoptotic cell death pathway; their action mechanism is believed to be based on reactive oxygen species (ROS) activity^50^. Hydroxyl radicals (OH^•^) are the most reactive form among the ROS, and induces the photo-cytotoxic effect upon their contact with cellular membranes. We used TA probe to detect the generation of OH^•^ by the NPs (Fig. 8e). Both USIO and MAg NPs are able to generate OH^•^, particularly under the irradiation. MAg NPs generated OH^•^ with higher efficiency than USIO NPs, which is in a good agreement with photo-cytotoxicity results. However, the ROS generation is one of the photo-cytotoxicity mechanisms, therefore further studies allowing for the detection of total ROS generation and also singlet oxygen generation will be pursued.

## Discussion

**Figure 9:**
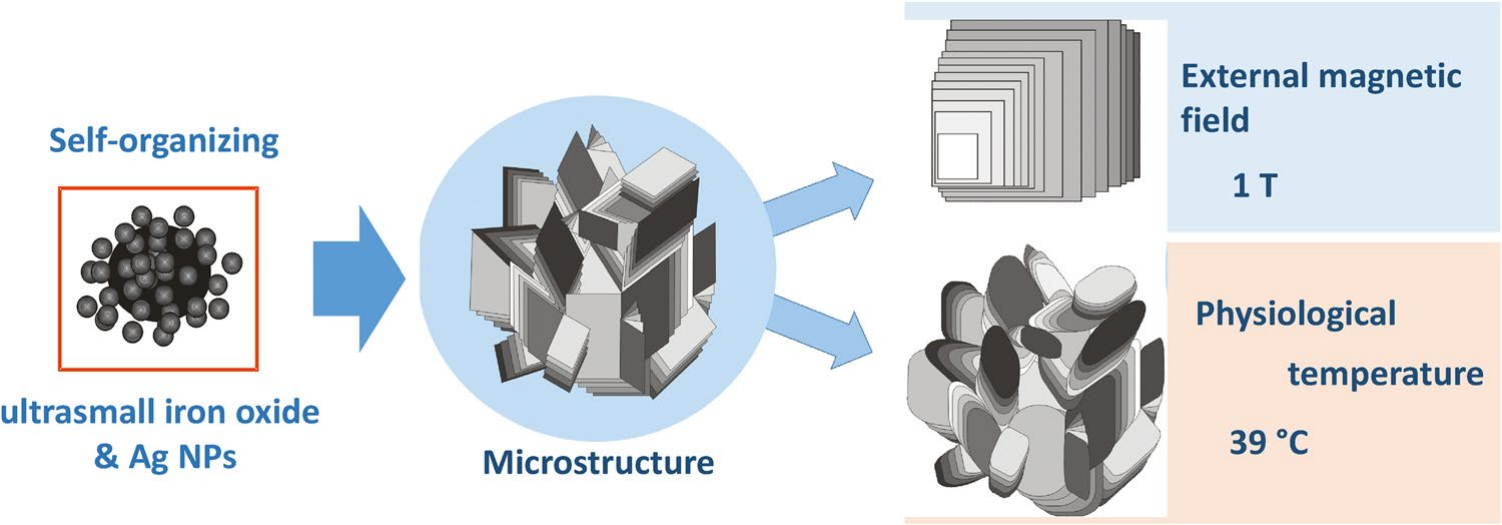
Scheme of the USIO and MAg NPs hydrocolloids microstructure transformation under the action of magnetic field and increased temperature.

In this work, self-organizing MAg and USIO NPs were synthesized by co-precipitation technique using ginger extract as a capping agent. These NPs form highly-ordered microstructure in hydrocolloids and are superparamagnetic by nature. As known, magnetic moment in USIO NPs is not stable due to disordered surface layer and small apparent magnetic size (*d*_0_ ~ 0.56 nm)^43^, that makes impossible to manipulate them by using magnetic field. Our study showed that magnetic field applied for several hours to USIO and MAg hydrocolloids influenced their microstructure without changing its structural elements (lamellae domains) but providing uniformity (schematic illustration on Fig. 9). This phenomenon may be explained by USIO NPs spins ordering that forces the microstructure reorganization. The interparticle forces in the hydrocolloids (van der Waals, electrostatic repulsion, magnetic dipole-dipole interaction, H-bonding, etc.) are strong enough to keep microstructure order. Applying magnetic field induces magnetic moments in iron oxide NPs, insufficient for their separation but sufficient for reorganization of diamagnetic Ag NPs within the microstructure. This process needs time due to the necessity of overcoming the resistance of NPs environment. Besides microstructure, the magnetic field affected also fluorescence emittance, conductivity and electro potential values of hydrocolloids.

Applied physiological temperature affected differently NPs diluted dispersions and hydrocolloids. In diluted dispersions the dispersibility of NPs was generally improved that may be explained in terms of enhancement of repulsive interparticle interactions and chaperone-like activity of BSA molecules (for NPs dispersions in BSA). While the hydrocolloids underwent changes in microstructure: its elements became rough and, instead of regular geometric shapes, roundish shapes appeared (see Fig. 9). Besides microstructure, the physiological temperature affected also conductivity of hydrocolloids.

Improved phototoxic properties of USIO and MAg NPs under the action of magnetic field are attributed to (i) reorganization of the microstructure under the action of magnetic field and (ii) generation of ROS (OH^•^ radicals). Reorganization of the microstructure probably induced surface electron transfer between NPs improving generation of ROS and surface plasmon resonance effect. This result is in agreement with previous findings in which aggregation of NPs has been shown to impact the fluorescence emittance; aggregated metallic NPs displayed significantly enhanced photoluminescence compared to not aggregated ones^51,52^. For future medical application, MAg NPs may be also excited by longer wavelengths (780 nm, two-photon) that will allow the light to penetrate deeper and treat larger tissue areas.

The observed changes in hydrocolloids microstructure of USIO and MAg NPs under the action of aforementioned factors showed that microstructure is of great significance for future NPs application due to the its impact on NPs properties. This ability makes them a promising candidate for preparation of scaffolds, films and gels with unique properties for technical and biomedical application.

## Methods

The synthesis of MAg NPs were performed via co-precipitation technique described in^22^. Ginger route extract was used as a capping agent. The synthesis of solely silver (Ag) NPs and ultrasmall iron oxide (USIO) NPs were performed using the above mentioned procedure but without the addition of iron salts and silver nitrate, respectively. USIO and MAg NPs were found to form stable water dispersions. At high concentrations, the hydrocolloids turned into thixotropic hydrogel in time (see Supplementary Fig. S11). Throughout the article, the samples were investigated in different states: as a powder (for XRD, SEM EDS, FTIR, fluorescence emittance), as a dispersion (with low concentrations, ~1 mg/ml) (for UV-Vis, Zeta-sizer, DLS, etc.) and as a hydrocolloid (68 ± 2 mg/ml) (for fluorescence emittance, cryo-SEM, etc.). For optical microscopies measurements, the NPs water dispersions (optical density (OD) ≤1) were used. For detailed description of methods used, see Supplementary Information.

## Electronic supplementary material

Supplementary Information
