# Supplementary material for: Silver and ultrasmall iron oxides nanoparticles in hydrocolloids: effect of magnetic field and temperature on self-organization
Source: Sci Rep. 2018 Mar 6;8:4041. doi: 10.1038/s41598-018-22426-2 (PMC5840429; doi:10.1038/s41598-018-22426-2)
Supplement: Supplementary file 1 — Supplementary Information [file 41598_2018_22426_MOESM1_ESM.pdf]

SUPPLEMENTARY INFORMATION

Silver and ultrasmall iron oxides nanoparticles in hydrocolloids: effect of magnetic field and temperature on self-organization

Olena Ivashchenko[a]*, Barbara Peplinska[a], Jacek Gapinski[b], Dorota Flak[a], Marcin Jarek[a], Karol Załęski[a], Grzegorz Nowaczyk[a], Zuzanna Pietralik[c] and Stefan Jurga[a]

[a] *NanoBioMedical Center, Adam Mickiewicz University, 61614 Poznań, Poland*

[b]*Department of Molecular Biophysics, Adam Mickiewicz University, 61614 Poznań, Poland*

[c]*Department of Macromolecular Physics, Adam Mickiewicz University, 61614 Poznań, Poland*

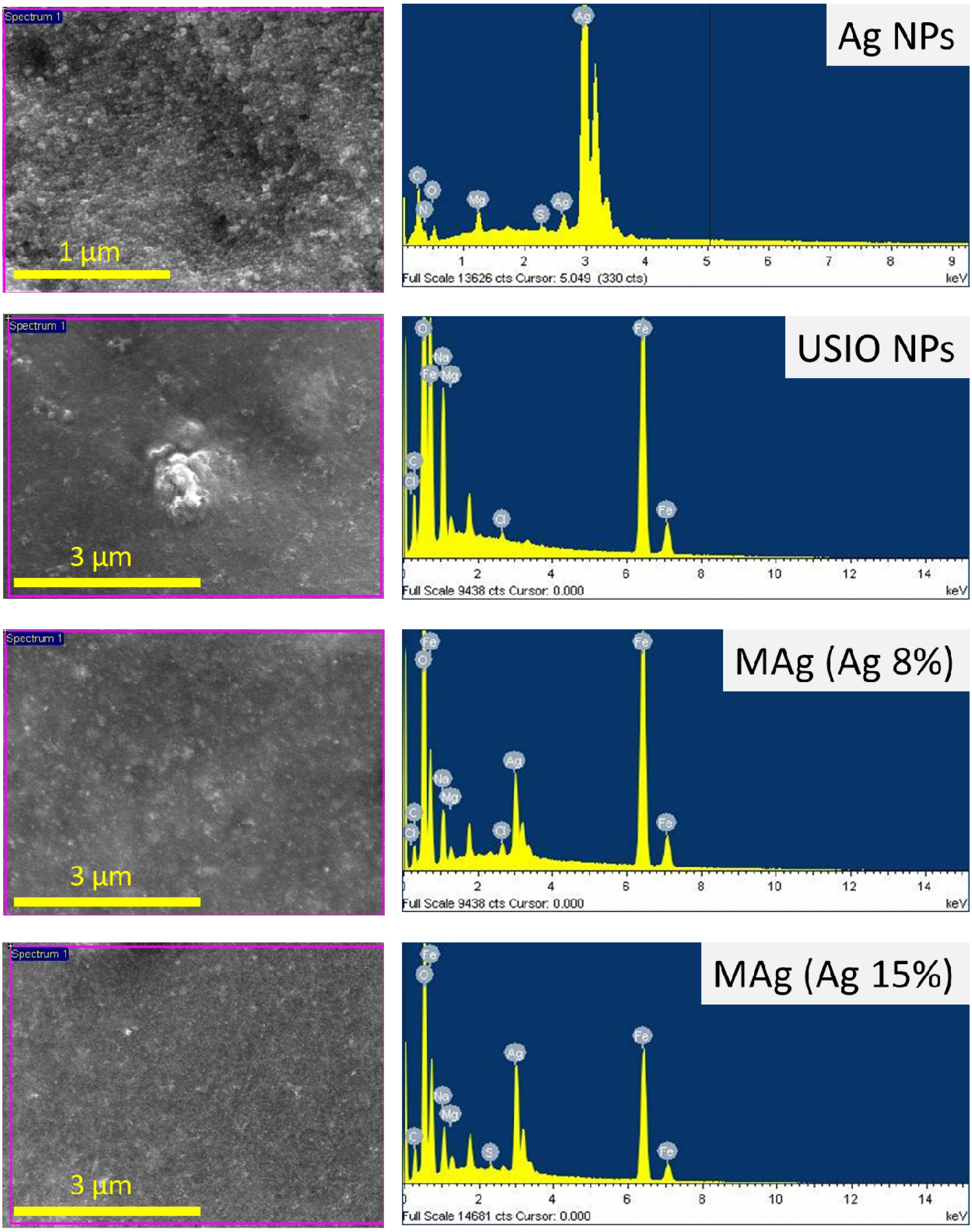

Fig. S1. The results of SEM EDS measurements for Ag, USIO and MAg NPs (powders).

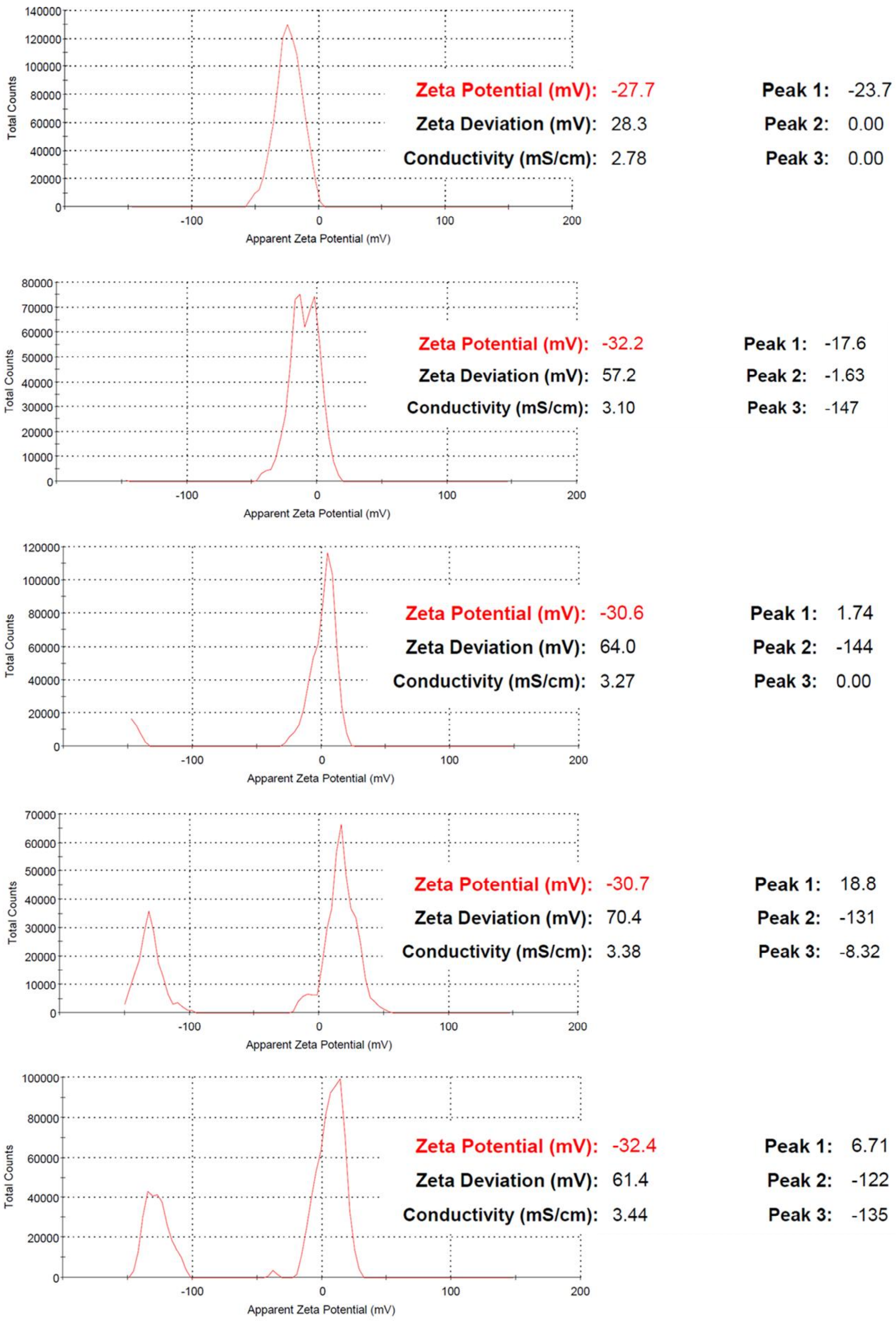

Fig. S2. The example of serial zeta-potential measurements for MAg NPs dispersions that showed dynamic changes in charge distribution.

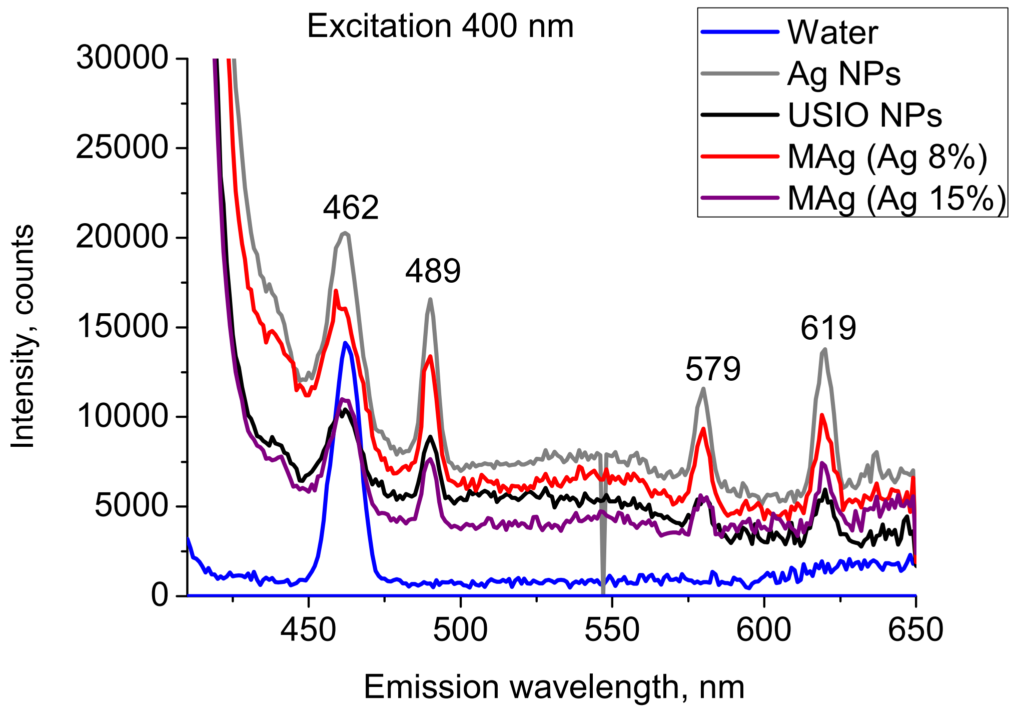

Fig. S3. Fluorescence emission spectra of diluted dispersion of USIO, Ag and MAg NPs (optical density (OD) <1) at excitation wavelengths 400 nm.

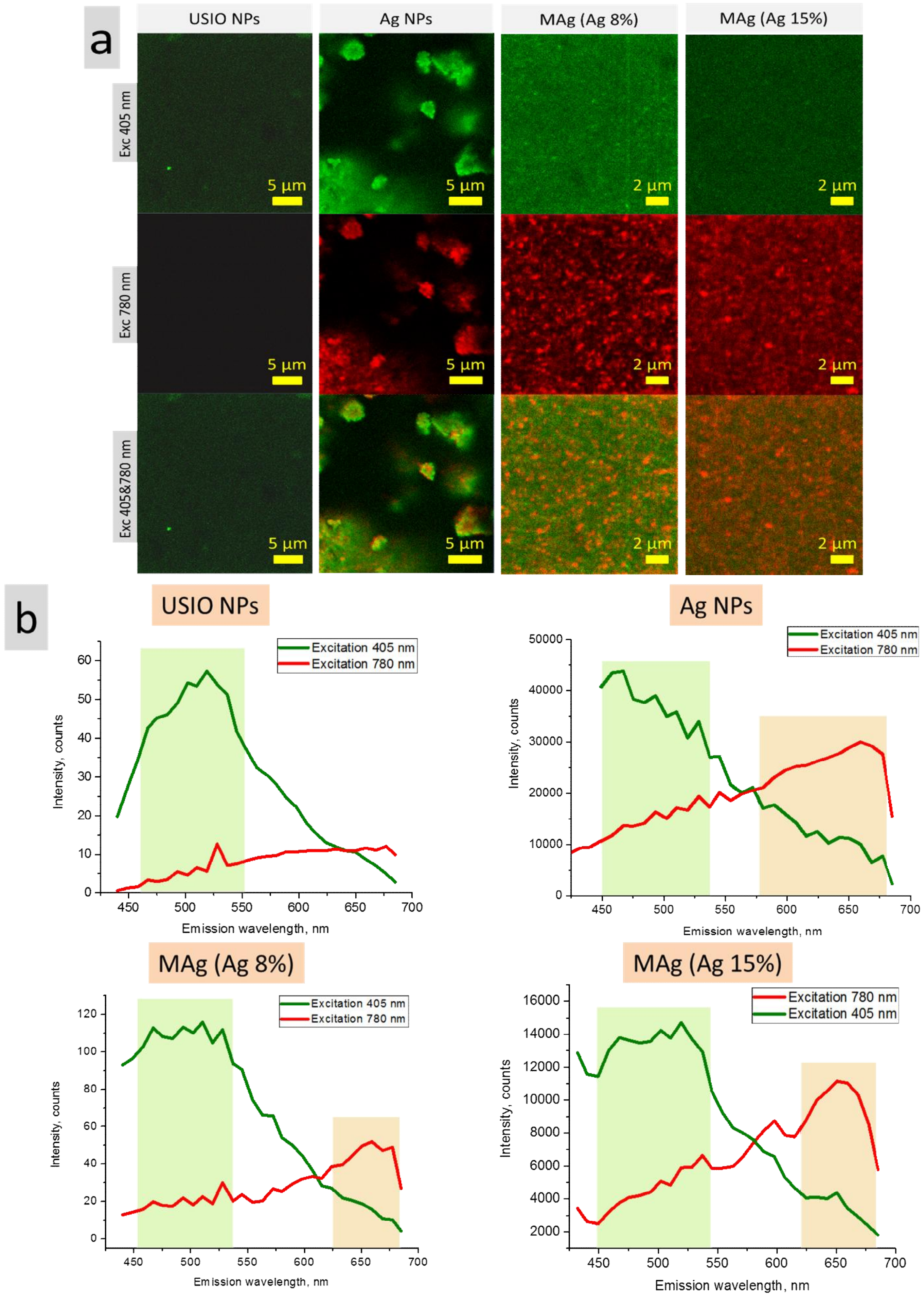

Fig. S4. Fluorescence images (a) and emission spectra (b) of USIO, Ag and MAg NPs hydrocolloids.

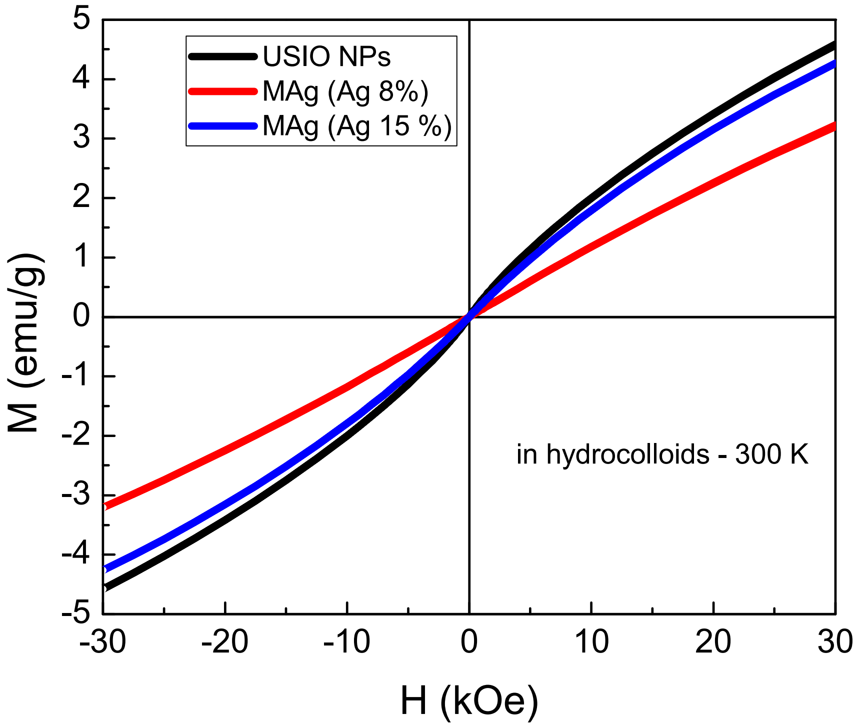

Fig. S5. Magnetization curves for USIO and MAg NPs hydrocolloids calculated relatively amount of powdered NPs.

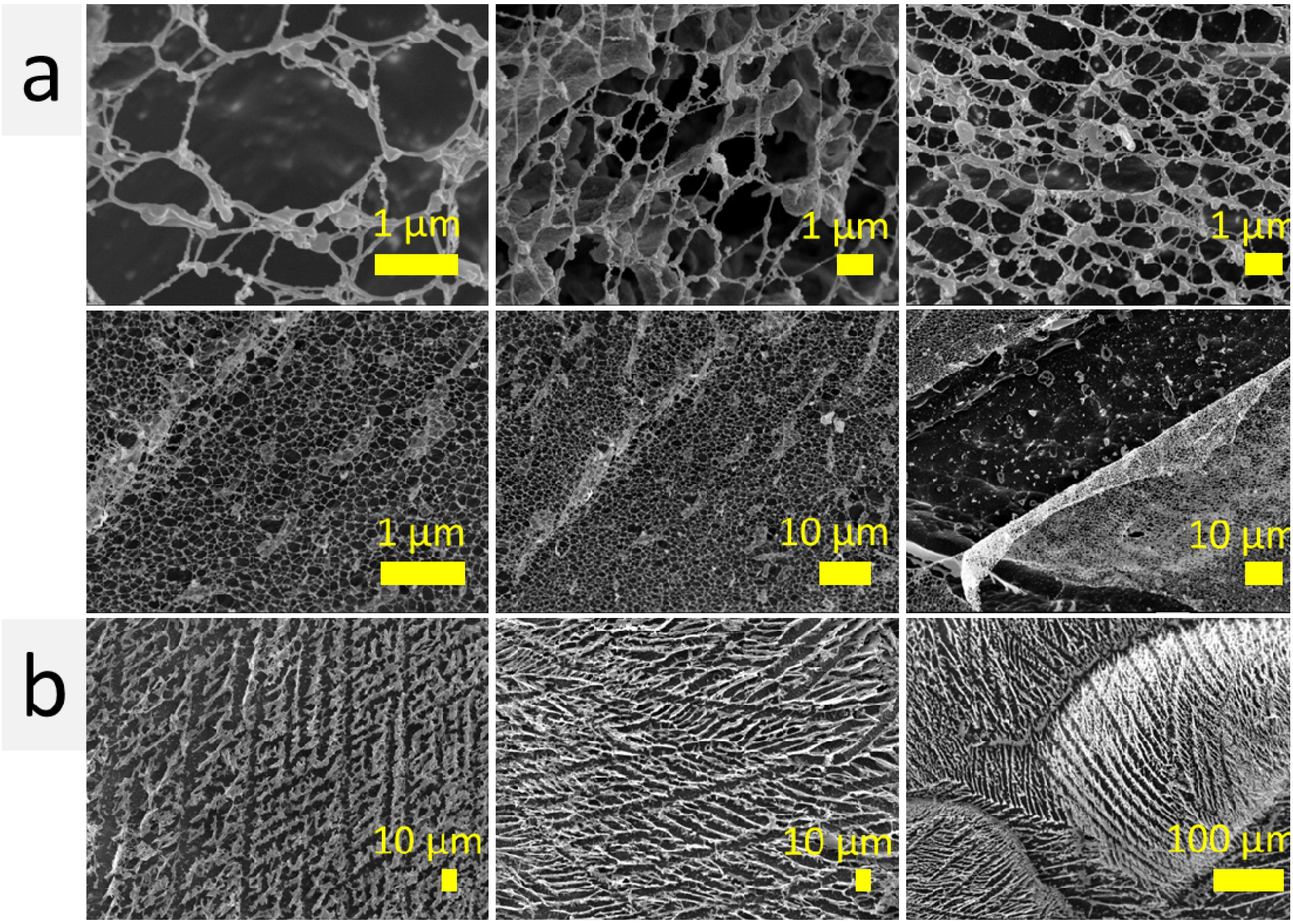

Fig. S6. Cryo-SEM images demonstrating surface structures of hydrocolloids which are typical for all the samples: net structure (a) and parallel stripes (b).

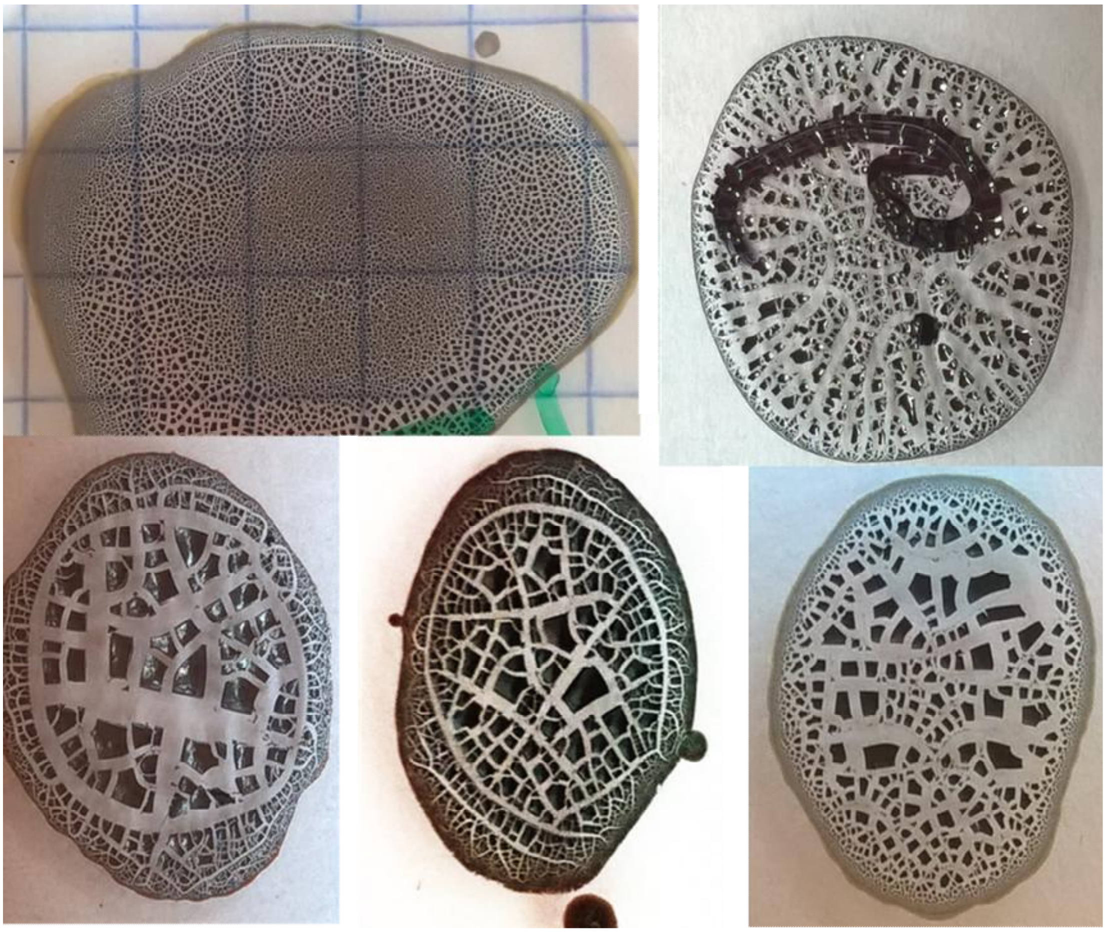

Fig. S7. Coffee ring effect of MAg hydrocolloids evaporated at 50 °C.

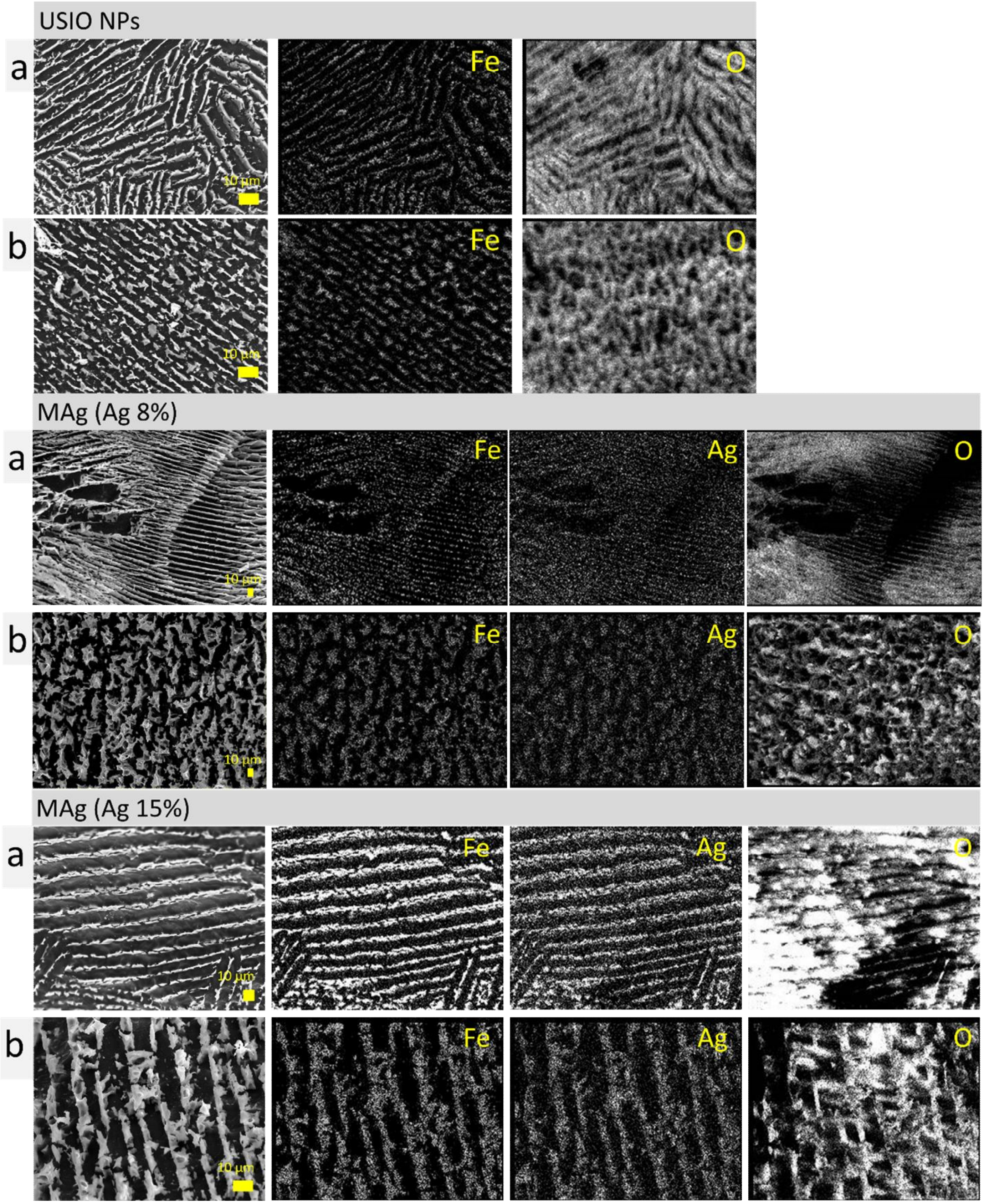

Fig. S8. Cryo-SEM EDS images of the hydrocolloids of USIO and MAg NPs without (a) and with (b) exposition under permanent magnetic field.

Table S1. Conductivity and electro potential of USIO and MAg NPs hydrocolloids before and after exposition in magnetic field

	Conductivity, mS/cm		Potential, mV	
	Before	After magnetic field	Before	After magnetic field
USIO NPs	6.8±0.2	7.1±0.2	-320.0±3.0	-320.2±3.3
MAg (Ag 8%)	4.5±0.3	4.7±0.2	-199.5±2.4	-210.6±2.0
MAg (Ag 15%)	3.2±0.2	3.4±0.2	-244.3±2.6	-252.0±2.1

Dynamic light scattering measurements

When interpreting the DLS data obtained in samples containing charged particles at relatively high concentration one should keep in mind that the measured diffusion coefficient values used to calculate the hydrodynamic radii are strongly biased by the interaction effects which in most cases place those apparent radii to the lower values than the real ones. For example, the apparent radius of pure BSA measured at finite concentration was only 1.0 nm at 20°C and 0.68 nm at 39°C (Fig. S9). In the case of simple colloidal suspensions the true size distribution is obtained by extrapolation of the results obtained at finite concentration to zero concentration limit. However, in the case of interacting multicomponent mixture such procedure is not possible and only apparent size distributions can be obtained. Nevertheless, some comparison between colloidal stability and interactions in different conditions can be made.

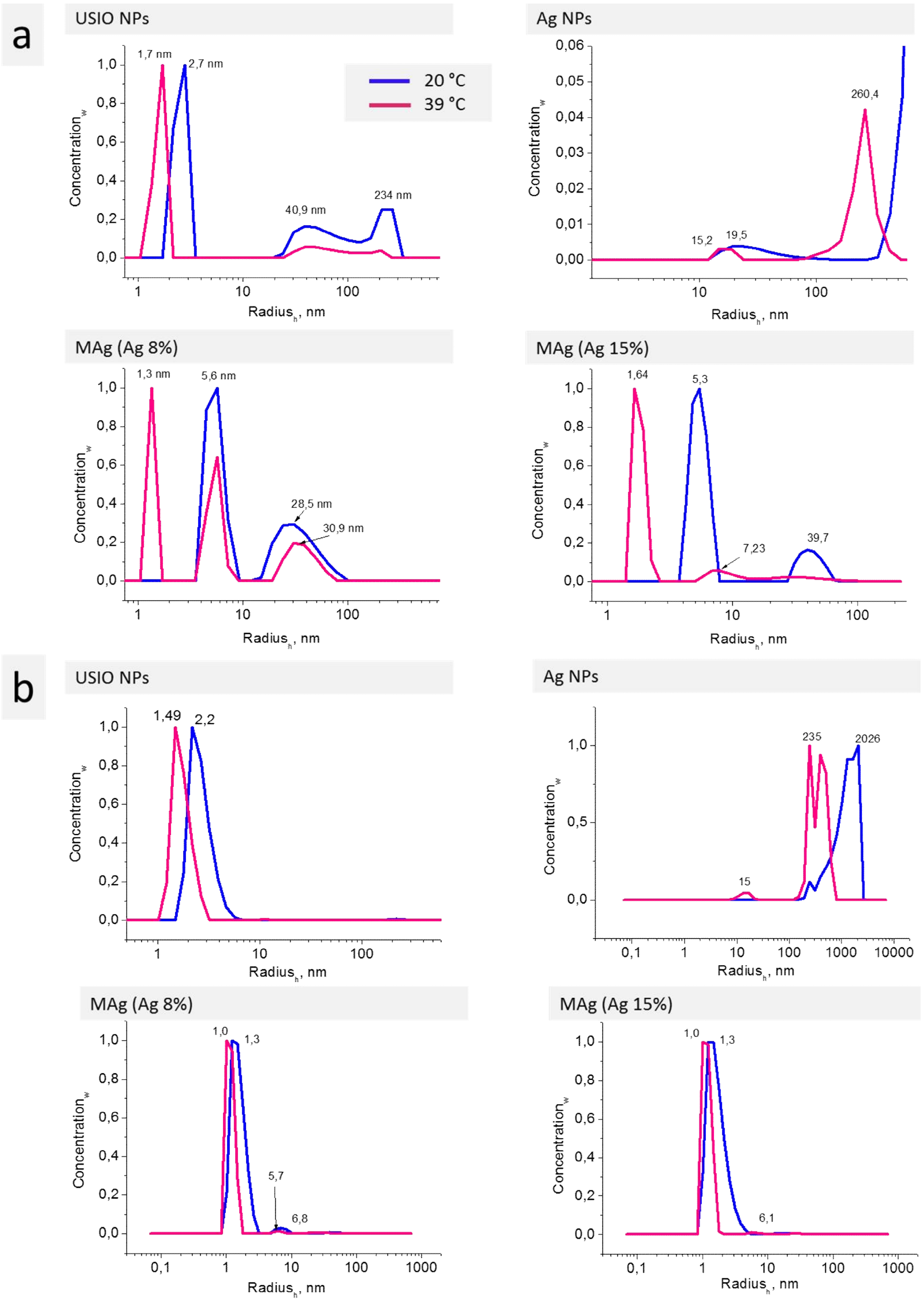

Fig. S9. Apparent size distributions obtained by means of DLS for the dispersions of the USIO, Ag and MAg NPs at 20 and 39 °C in water (a) and BSA solution (b); the data are presented as weight concentration distributions within the approximation $M_w \propto R^3$.

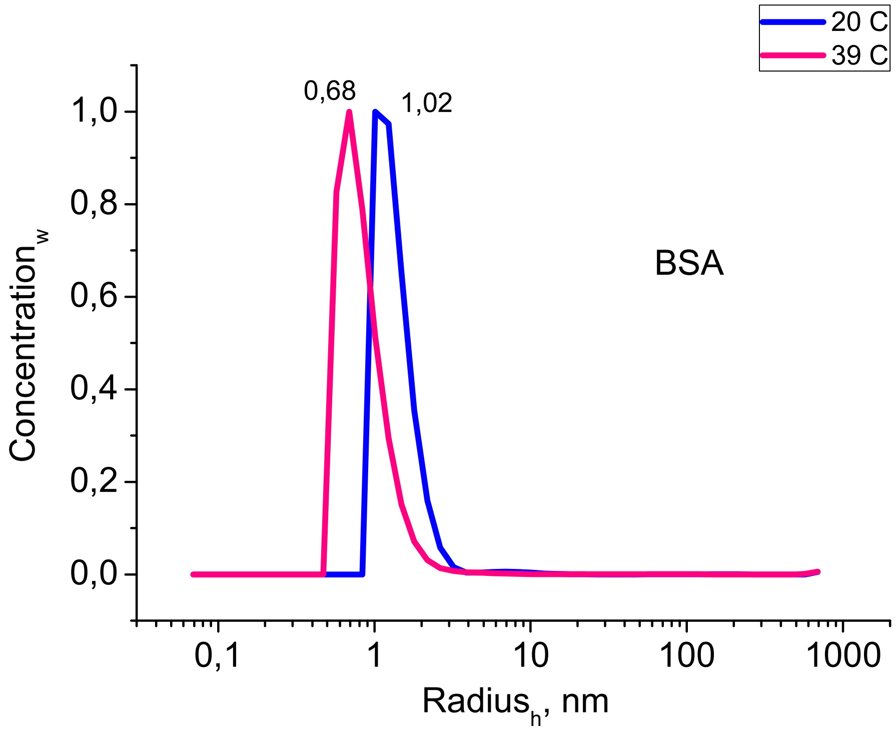

Fig. S10. Apparent hydrodynamic size of BSA protein molecules at 20 and 39 °C according to DLS measurements.

Table S2. Conductivity and electro potential of USIO and MAg NPs hydrocolloids at room (21 °C) and increased (39 °C) temperature

	Conductivity, mS/cm		Potential, mV	
	21 °C	39 °C	21 °C	39 °C
USIO NPs	7.8±0.2	10.5±0.5	-323.5±4.3	-312.0±4.0
MAg (Ag 8%)	5.7±0.2	7.4±0.2	-199.5±3.3	-205.5±2.5
MAg (Ag 15%)	3.8±0.3	5.2±0.2	-235.3±2.6	-243.6±3.4

Methods

Materials

The analytical grade reagents of iron (III) chloride hexahydrate ($FeCl_3 \cdot 6H_2O$), iron (II) chloride tetrahydrate ($FeCl_2 \cdot 4H_2O$), silver nitrate ($AgNO_3$), ammonium hydroxide (NH_4OH), hydrochloric acid (HCl), bovine serum albumin (BSA) and glucose were purchased from Sigma-Aldrich and used without further purification protocols. BSA water solution (1 wt%) was used throughout the experiments. Reagents for biological tests (phosphate buffer saline PBS), agar, formaldehyde, ethanol, TRITC-Concanavalin A) were also purchased (Sigma-Aldrich, LifeTechnologies) and used as received. Ultrapure water (resistivity > 17 MΩcm) from a GZY-P10 water system was used throughout the experiments.

MAg synthesis

The synthesis of MAg NPs were performed via co-precipitation technique described in [22]. Ginger route extract was used as a capping agent. To prepare ginger rhizome (*Zingiber officinale*) extract, a piece of ginger rhizome (250 g) was washed thoroughly with distilled water and then cut into small pieces. Chopped ginger rhizome was kept in a water-ethanol solution (250 ml, 1:1 ratio) for 5 days (room temperature, dark place). Then, supernatant was vacuum filtered (Whatman filter paper) and stored in refrigerator (4 °C). The dry residue concentration of ginger extract (dried at 50 °C) was 13.6 mg/ml.

In typical synthesis, 198.7 mg of $FeCl_2 \cdot 4H_20$ and 540.38 mg of $FeCl_3 \cdot 6H_2O$ were solved in water (20 ml) and mixed with ginger extract (10 ml). Simultaneously, $AgNO_3$ (40 or 80 mg) was solved in water (10 ml) and mixed with ginger extract (10 ml). The latter mixture was added dropwise to the iron salts solution. Then, NaOH solution (25 %, 12 ml) was added dropwise under rigorous stirring; the reaction mixture immediately turned black. The reaction mixture was heated to 85 °C for 1.5 h. When cooled, the deposit was washed with water (following centrifugation, 24000 rpm, 30 min).

The synthesis of solely silver (Ag) NPs and ultrasmall iron oxide (USIO) NPs were performed using the above mentioned procedure but without the addition of iron salts and silver nitrate, respectively.

USIO and MAg NPs were found to form stable water dispersions. At high concentrations, the hydrocolloids turned into thixotropic hydrogel in time (Fig. S11). Throughout the article, the samples were investigated in different states: as a powder (for XRD, SEM EDS, FTIR, fluorescence emittance), as a dispersion (with low concentrations, ~ 1 mg/ml) (for UV-Vis, Zeta-sizer, DLS, etc.) and as a hydrocolloid (68±2 mg/ml) (for fluorescence emittance, cryo-SEM, etc.). For optical microscopies measurements, the NPs water dispersions (optical density (OD) ≤1) were used.

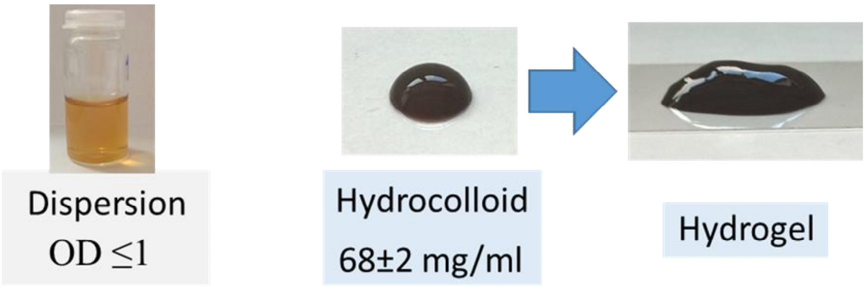

Fig. S11. Photos of typical USIO and MAg NPs dispersion, hydrocolloid and hydrogel.

Characterization techniques

Physicochemical techniques

X-ray diffraction (XRD) studies were conducted on an Empyrean diffractometer (PANalytical), using Cu Kα radiation (1.54 Å), a reflection-transmission spinner (sample stage) and PIXcel 3D detector, operating in the Bragg–Brentano geometry. The °2Theta scans were recorded at angles ranging from 10 to 95° with a step size of 0.007° and continuous scan mode. XRD measurements were performed on powdered samples.

Elemental analysis of the samples was carried out by means of the energy dispersive microanalysis (EDS) mode of an X-ray equipped JEOL 7001 F Scanning Electron Microscope (SEM) (SEI detector, 15 kV accelerating voltage) using three parallel runs at magnification ×20000.

Transmission Electron Microscopy (TEM) measurements and elemental analysis were performed using a JEM-ARM-200F High Resolution Transmission Electron Microscope (accelerating voltage of 200 kV) equipped with dispersive X-ray spectrometer.

Ultraviolet–visible spectroscopy (UV-Vis) measurements were performed using a Lambda 950 spectrophotometer (Perkin Elmer). Size distribution and zeta-potential of nanoparticles were measured on the ZetaPlus Analyser (Malvern). The excitation spectra, emission spectra and lifetime of the samples were obtained by FluoroSENS Spectrophotometer (Gilden Photonics).

Dynamic light scattering (DLS) apparatus consisted of a solid state laser 532 nm operating with a power of 100 mW, an ALV Goniometer thermostated with an accuracy of 0.1 °C and ALV7000 digital correlator (ALV GmbH, Germany). Two avalanche photodiodes (Perkin Elmer SPCM-AQR) were used in the pseudo cross-correlation mode in order to provide proper statistics in the short-time range of the correlation functions. Measured correlation functions were analyzed by means of the regularized fitting routine (CONTIN-like) built in the ALV software (ver. 3.0).

The Fourier transform infrared (FTIR) transmittance spectra were obtained using a Tensor 27 (Bruker Optics) spectrometer equipped with a global source and MCT detector. Samples were prepared

using KBr as a matrix material, and mixed in proportions of 1 mg of sample to 200 mg KBr. Preparation of ginger dry residue was performed as follows: 20 ml of ginger rhizome extract was evaporated at 50 °C and then dry residue of the extract was collected and used for pellet fabrication.

The magnetization measurements have been performed using a MPMS-XL SQUID magnetometer. Zero-field-cooled (ZFC) and field-cooled (FC) magnetization curves were measured at 100 Oe with temperature varying from 2 to 350 K. The magnetization dependence on the magnetic field (M-H curves) was measured at 5 K, 100 K and 300 K at magnetic field varying between ±30 kOe.

The microstructure of USIO and MAg NPs hydrocolloids was examined by cryogenic scanning electron microscopy (cryo-SEM) method (accelerating voltage 5 kV). The samples were cryo-fixed by plunging them into sub-cooled nitrogen (nitrogen slush) close to the freezing point of nitrogen at –210 °C. Cryo-SEM elemental mapping was performed using the energy dispersive microanalysis (EDS) mode of an X-ray equipped SEM (accelerating voltage 15 kV). As cryo-SEM technique is relatively novel, we would like to mention two features observed in the course of the measurements. First, regular observation of radially-directed areas on the samples (Fig. S12a). We related these areas to processes of compression and retraction of hydrocolloids due to their rapid freezing. That is why these radially-oriented areas were not taken into account in our experiments. Second, we noticed that the duration of sublimation is crucial for microstructure observation. To optimize it, the MAg hydrocolloids underwent sublimation for 20, 30 and 40 min. We found that 20 and 30 min are insufficient for free water removal and the microstructure was still covered by a layer of bulk water (Fig. S12b). The optimal sublimation time for the hydrocolloids without exposition to magnetic field was found to be 40 min, including 15 min for sublimation of the cleaved part of the sample. However, these conditions were found to be insufficient for the hydrocolloids treated with magnetic field because their microstructures were still hidden under water layer, so the sublimation time of cleaved part was increased from 15 to 30 min.

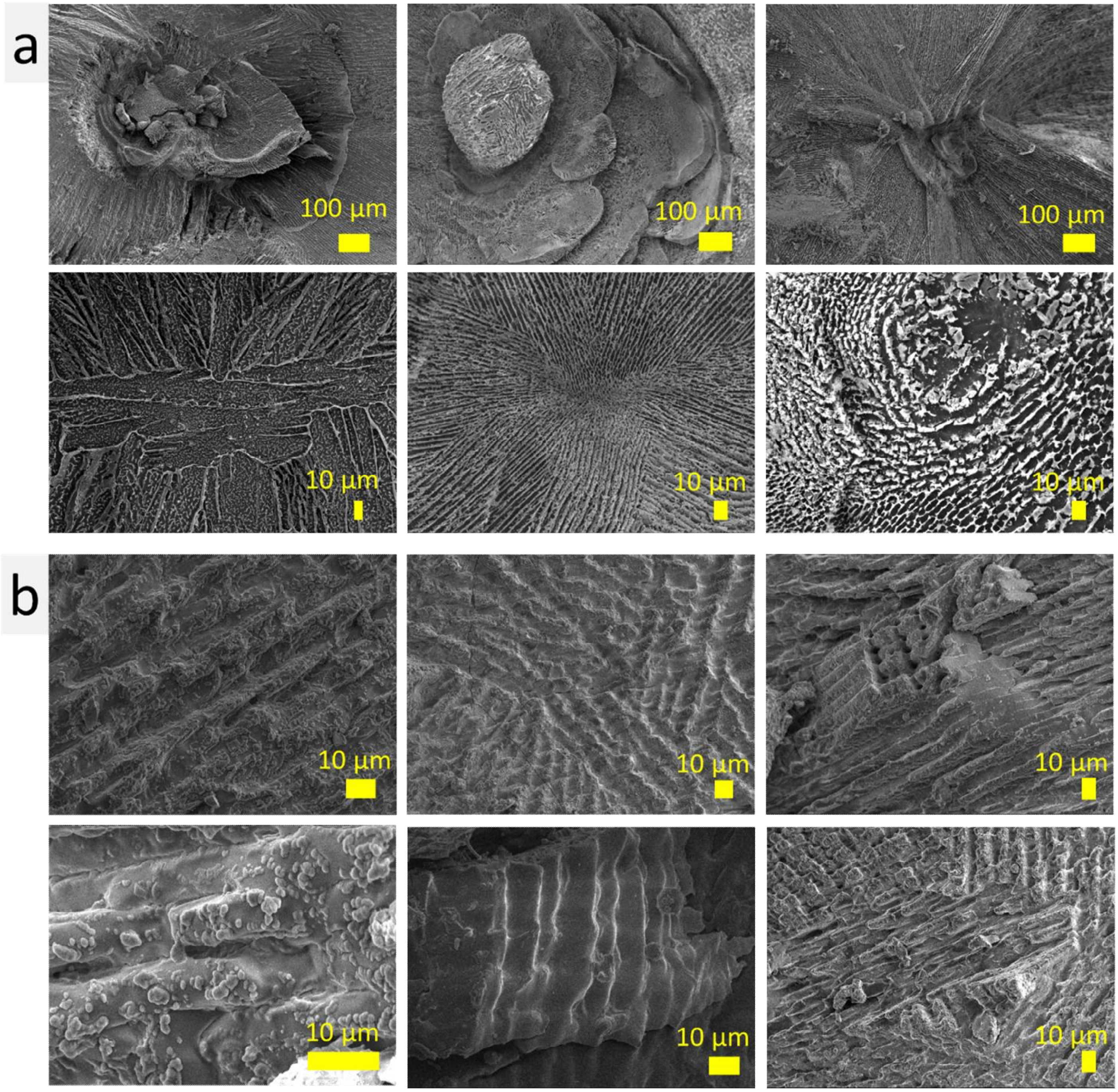

Fig. S12. Cryo-SEM images: radially-directed areas on the USIO and MAg NPs hydrocolloids, appeared due to retraction at rapid freezing, observed during measurements (a); a sample with insufficient sublimation time: the microstructure of hydrocolloid is covered by water layer (b).

To find out the influence of an external static magnetic field on the hydrocolloids microstructure, the duration of exposition to the permanent magnetic field (1 T, 21 °C) was varied. We checked the morphology of hydrocolloids before and after the exposition to magnetic field using cryo-SEM technique. The duration of exposition was varied from 30 min to 20 h. More than 1200 images were taken and analyzed focusing on the inner structure of the samples. Three independent experiments for each sample with two parallel probes were performed.

Fluorescence measurements were performed by means of a laser scanning microscopy system LSM 780 (Zeiss, Germany) equipped with a femtosecond tuneable infrared laser for two-photon excitation. In

order to reveal the influence of magnetic field on fluorescence emittance of hydrocolloids, the samples were exposed to magnetic field for 4 h (1 T, 21 °C).

Conductivity and electro potential measurements of hydrocolloids were performed before (21 °C) and after exposition to magnetic field (1 T, 5 h, 21 °C), and after exposition at increased temperature (39 °C, 4 h) using pH/Conductometer CPC-505 (Elmetron).

Phototoxicity assay

Prior to the photo-cytotoxicity activity studies, a stock suspension of prepared nanoparticles in sterilized deionized water (18.2 MΩ·cm at 25°C) was prepared by sonication and further by UV sterilization. Suspensions with the final required concentration of 100 μg/ml were prepared by the dilution of the stock solution in cell culture medium (DMEM) and then by sonication before cellular exposure. As-prepared suspensions were added onto four sets of previously prepared HeLa adherent cells in a 96-well plate at the concentration of 0.8×10^4 cells/ml and then co-incubated in standard cell culture conditions in incubator for 24 h before further treatment. Two sets were additionally positioned on the Mega Magnetic Plate (OZ Biosciences) in order to ensure an exposure to the external magnetic field. Two of as-prepared sets (not exposed and exposed to the magnetic field) were further used for the experiment with no irradiation and another two for the experiment under light irradiation. The sets also included negative control, and positive control (DMSO), where no nanoparticles and 50 v/v% dimethyl sulfoxide in deionized and sterilized H_2O were added, respectively. Afterwards, as-prepared cells were washed with PBS, then added with PBS as a medium and finally irradiated using near-visible light exposure (405 nm with power density of 2.30 mW/cm^2, 635 nm with power density of 1.35 mW/cm^2) for 40 minutes. After irradiation the medium was exchanged back to cell culture medium and cells were incubated in standard cell culture condition for 24 h prior to viability assay. The cell viability, in terms of the metabolic activity, was assessed using the In Cell Analyser apparatus (GE Healthcare) and Live/Dead viability Kit (Life Technologies) employing fluorescent dyes, calcein-AM and ethidium homodimer (EthD-1), for staining of live and dead cells, respectively. The tests were carried out in triplicate. The fluorescent images were acquired from 20 fields from each well (objective Nikon 20x), and then subjected to the statistical analysis by employing In Cell Developer Toolbox software.

Detection of the generated hydroxyl radicals

The ability of prepared USIO, Ag and MAg NPs to generate hydroxyl radicals ($OH^•$) was determined with the use of the terephtalic acid (TA) fluorescence probe. TA is known to react with $OH^•$ resulting in generation of TAOH, which emits fluorescence at arr. 427 nm on the excitation of 312 nm. For the measurements purpose the aqueous solution containing 10 mM NaOH and 0.5 mM TA was prepared. Then prepared NPs were suspended in this reaction solution in the concentration of 100 μg/ml and given to the irradiation of 405 nm (power density of 2.30 mW/cm^2) for 45 in a dark box and under continuous stirring. Fluorescence spectra of the supernatant liquid were measured with the FluoroSENS Spectrophotometer (Gilden Photonics).
